# Disposable all-printed electronic biosensor for instantaneous detection and classification of pathogens

**DOI:** 10.1038/s41598-018-24208-2

**Published:** 2018-04-12

**Authors:** Shawkat Ali, Arshad Hassan, Gul Hassan, Chang-Ho Eun, Jinho Bae, Chong Hyun Lee, In-Jung Kim

**Affiliations:** 1 Department of Ocean System Engineering, Jeju National University, 102 Jejudaehakro, Jeju, 63243 South Korea; 2 Subtropical/tropical Organism Gene Bank, Jeju National University, Jeju, 63243 Republic of Korea; 3 Faculty of Biotechnology, College of Applied Life Sciences, Jeju National University, 102 Jejudaehakro, Jeju, 63243 South Korea; 4 Department of Electrical Engineering, National University of Computer and Emerging Sciences FAST, H 11/4, Islamabad, 44000 Pakistan; 5 Present Address: Department of Ocean System Engineering, College of Ocean Science, Jeju National University, 102 Jejudaehakro, Jeju-si, Jeju Special Self-Governing Province 63243 Republic of Korea

## Abstract

A novel disposable all-printed electronic biosensor is proposed for a fast detection and classification of bacteria. This biosensor is applied to classify three types of popular pathogens: *Salmonella typhimurium, and the Escherichia coli* strains JM109 and DH5-α. The proposed sensor consists of inter-digital silver electrodes fabricated through an inkjet material printer and silver nanowires uniformly decorated on the electrodes through the electrohydrodynamic technique on a polyamide based polyethylene terephthalate substrate. The best sensitivity of the proposed sensor is achieved at 200 µm teeth spaces of the inter-digital electrodes along the density of the silver nanowires at 30 × 10^3^/mm^2^. The biosensor operates on ±2.5 V and gives the impedance value against each bacteria type in 8 min after sample injection. The sample data are measured through an impedance analyzer and analyzed through pattern recognition methods such as linear discriminate analysis, maximum likelihood, and back propagation artificial neural network to classify each type of bacteria. A perfect classification and cross-validation is achieved by using the unique fingerprints extracted from the proposed biosensor through all the applied classifiers. The overall experimental results demonstrate that the proposed disposable all-printed biosensor is applicable for the rapid detection and classification of pathogens.

## Introduction

A rapid and low-cost detection and classification of bacterial contamination is important information in many practical applications such as food industry^1,2^. Food safety, in particular, is a critical global issue with public health implications because foodborne outbreaks can create health crises. The World Health Organization (WHO) defines foodborne illness as a disease caused by agents that enter into a body through food^3^. According to a 2015 WHO report, it has been estimated that 1 out of 10 people in the world have fallen ill after consuming contaminated food. 420,000 people have also died from foodborne illnesses, including 125,000 children under the age of 5^4^. In South Korea, it has been reported that the annual foodborne illness estimate is 3,361,385 people^5^. The foodborne pathogens are the main source of causing foodborne illness as they rapidly grow and create toxic effects in food^6^. For the reason, the proper identification methods of foodborne pathogens can provide a solution to prevent foodborne disease outbreaks. On the contrary, some of the bacteria are good for health, i.e., in yogurt, which need easy and exact classification of bacteria types for disease control. Most of the conventional methods rely on biological and biochemical processes for microbial identification^7^. These conventional methods are well-known as simple culture and colony counting methods that involve the counting of bacteria, morphological, enzyme linked immunosorbent, immunology based methods that involve the antigen-antibody interactions, and DNA analysis based quantitative Polymerase Chain Reaction (qPCR) or microarray methods. Although these methods are precise, they can give both qualitative as well as quantitative information of the observed microbes. These conventional methods have significant limitations in term of cost, special facilities, and a long procedural time^8–10^.

In terms of a detection time for bacteria, the previous methods mentioned should be accompanied with a fast prescreening, which helps provide a rapid detection^1,2^. Newly developed methods based on real time qPCR can detect rapidly and robustly compared to the other methods, but they are still restricted by time constraints as they require several hours as well as expensive apparatuses^8–12^. Similarly, another nondestructive technique using hyper-spectral imaging is a real time rapid method for identifying microbes^13–16^, but it is a high-cost technology due to it needing sensitive detectors. The food pathogens produce volatile compounds, including terpenes and alcohol, which have specific characteristic odors^17,18^. These bacterial species influence the amount of volatile compounds that are used as bio-markers for their identification^19,20^. In recent years, the electronic nose (E-Nose) with the MOS biosensor has been studied for real time monitoring of these characteristic odors. It was found that it could applied be applied to identify food pathogens because volatile compounds can change during bacteria growth^21–24^. However, the output signal of this sensor in the array is less reliable due to the cross interference caused by irrelevant gasses. The same problem occurs for all the other sensors in the array, which makes the output erroneous. The accuracy of the E-nose sensor improved with pattern recognition methods. However, the accuracy still remained below hundred percent^25–31^. In the impedance based bacteria sensors, most of the studies demonstrated bacteria detection methods by using the antibody to attract the bacteria towards the sensor’s electrode, but these methods need several hours to take place^32–35^. Secondly, the instrument cost is high and not available everywhere in developing countries due to their limited resources. In such countries, health centers, medicine, and food industries require low cost and disposable biosensors. To fulfil these requirements, a simple biosensor that has rapid detection and is low cost and environmentally friendly is essentially required.

**Figure 1:**
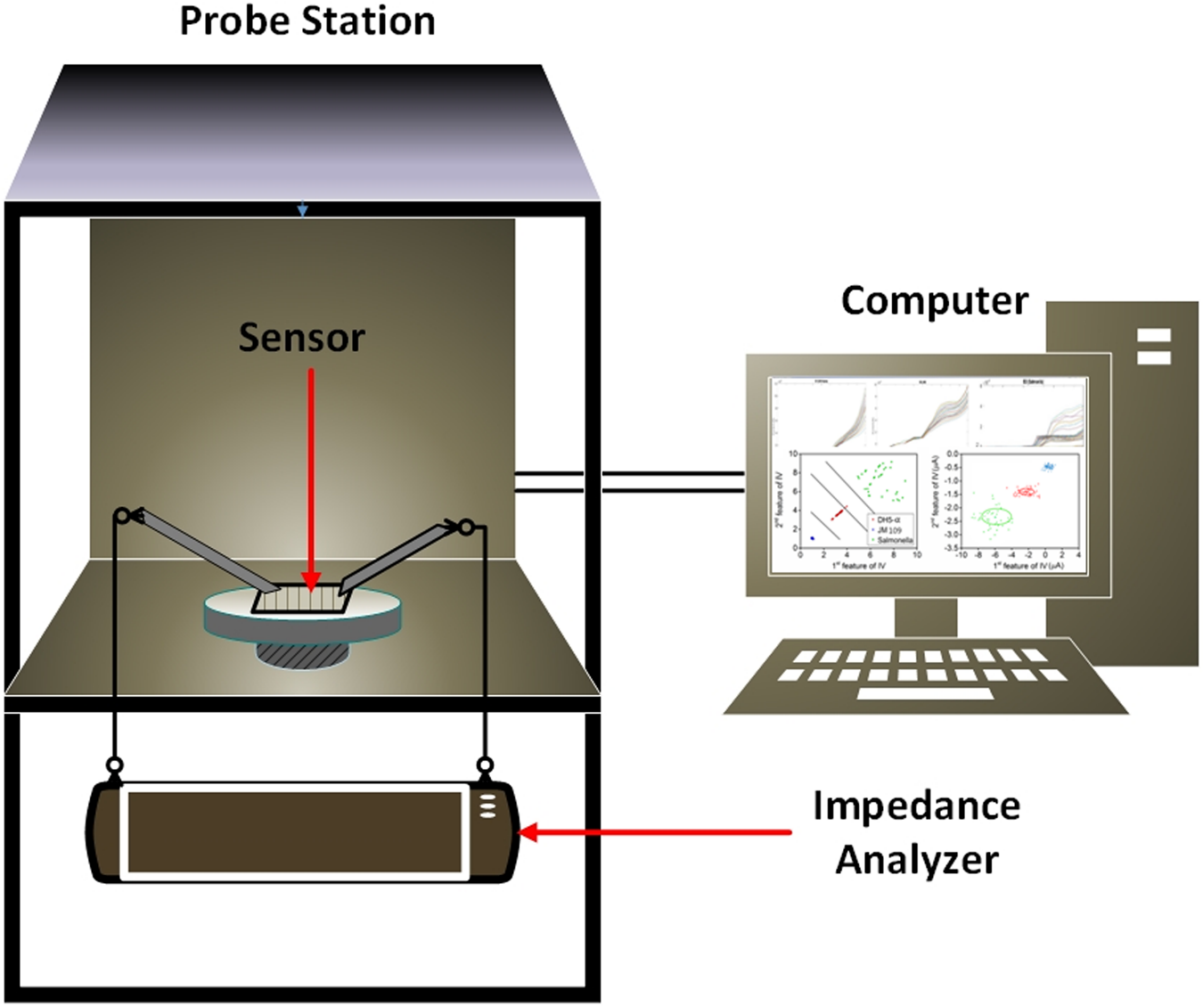
Schematic diagram of bacteria measurement and classification.

In this paper, we propose a novel impedance based biosensor that can detect three different types of bacteria, including *Escherichia coli*strains JM 109 and DH5-α, and *Salmonella typhimurium*. We verify this biosensor’s capability by using the various machine learning algorithms to classify them. Although both the *E. coli* JM 109 and DH5-α are gram-negative bacteria, which are not pathogens, these two strains have a similar phenotype to pathogen such as *E. coli* strain O157. These two strains were developed for laboratory molecular cloning. *Salmonella* is a genus of the rod-shaped gram-negative bacteria and facultative intracellular pathogen. *Salmonella* usually can cause self-limiting gastrointestinal diseases, and can be transmitted through the ingestion of contaminated food or water. These three bacteria organisms are quantitatively measured based on their impedance variation under the same conditions. The real-time impedance variation of the sensor for the three types of bacteria is measured through an impedance analyzer and the data are fed to a computer program for the classification, as shown in Fig. 1.

**Figure 2:**
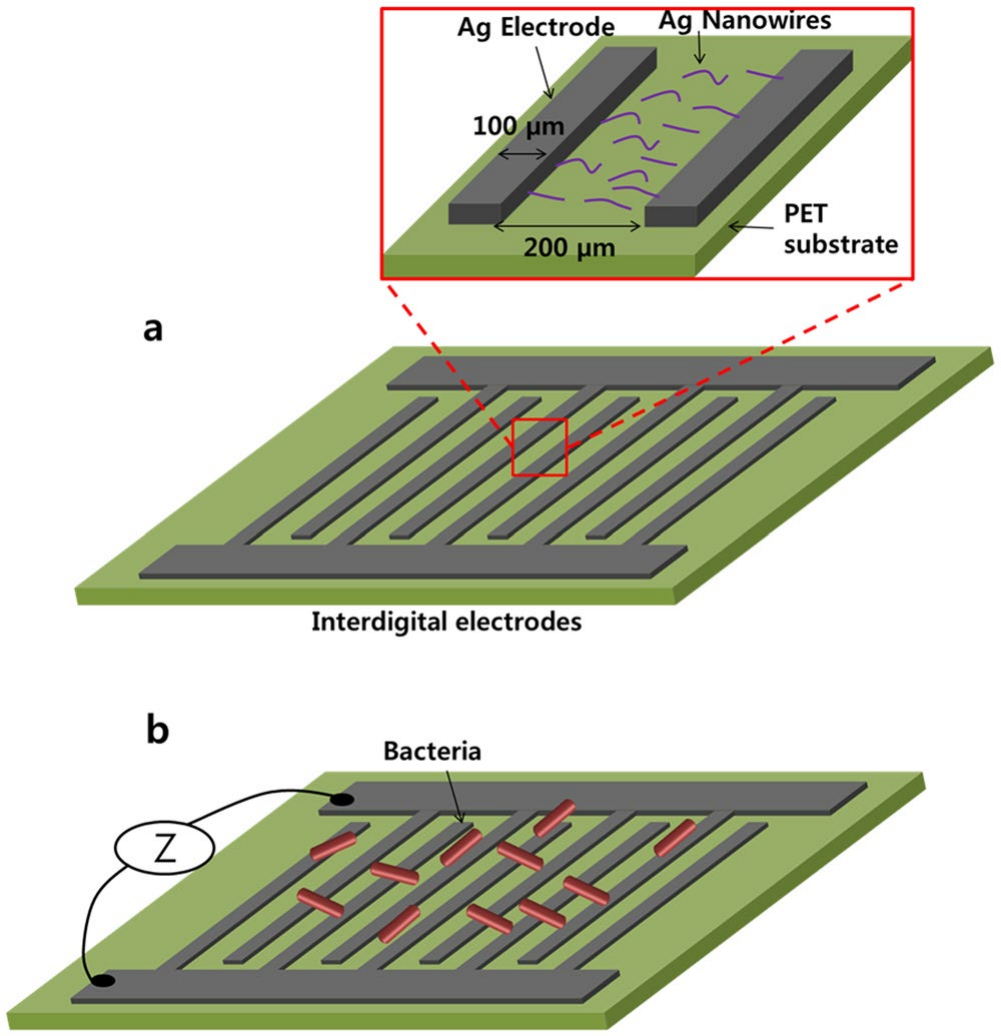
(**a**) Comb type electrodes on a PET substrate, the zoomed image shows Ag nanowires in the space between two electrodes. (**b**) Bacteria engaged with sensor that varies the impedance of the sensor.

The proposed biosensor consists of comb type silver electrodes fabricated on a transparent and flexible plastic polyethylene terephthalate (PET) substrate by utilizing a Fujifilm inkjet Dimatix material printer (DMP-3000). The inter-digital electrodes’ finger width is 100 µm and the separation between the fingers is 200 µm. To increase the sensitivity of electrodes for small bacteria detection, Ag nanowires are decorated over the electrodes through the electrohydrodynamic (EHD) printing technique, as shown in Fig. 2a. The Ag nano-wires facilitate the bacteria species in making an electrical connection between the finger electrodes. When the bacteria sample is dropped on the sensor, its impedance varies inversely against the bacteria concentration. Similarly, different types of bacteria of the same concentration can result in a slightly different impedance because of their chemical properties. For the same concentration (10^6^ CFU) of three types of bacteria, the impedance value is slightly different after 8 min of sample injection, which can lead to a rapid detection and classification of pathogens. Using the Agilent probe station, we measured the current voltage (I-V) values for three types of bacteria by applying a voltage sweep of ±2.5 V, and a small change in the measured data was processed by machine learning algorithms in Matlab R2015a to classify the type of bacteria. The pattern classification algorithms were used as linear maximum likelihood estimation (MLE), linear discrimination analysis (LDA), and non-linear back propagation neural network (BPNN) methods. In order to classify these bacteria, unique fingerprints, including the power, I-V curve, first, and second derivative of the I-V characteristics, were utilized to achieve 100% classification. These results show that the proposed low cost and disposable all printed biosensor can be a good candidate for the rapid detection and classification of food pathogens.

## Results

**Figure 3:**
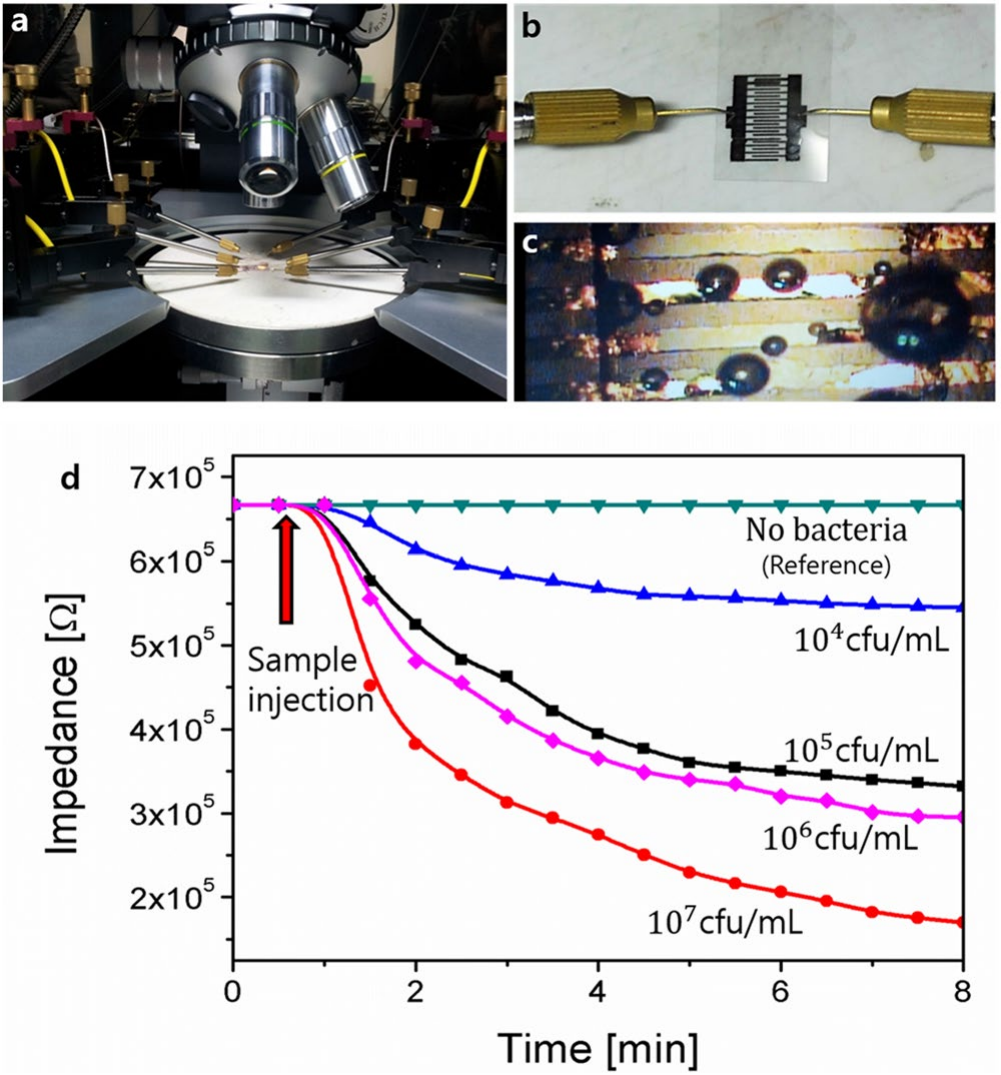
(**a**) Measurement setup of the bacteria sensor. (**b**) Bacteria sensor connected with probes for measurement. (**c**) Zoomed image of the sensor electrodes engaged with bacteria. (**d**) Impedance vs bacteria concentration curves of salmonella.

As shown in Fig. 2a, the optimized biosensor is based on silver inter-digital electrodes with 200 µm finger spacing and decorated with an Ag nanowire concentration of 30 × 10^3^/mm^2^ on a PET substrate (For further details about the sensor design, see section 1 in supplementary information.). The proposed biosensor measures impedance variation to detect and classify bacteria, which is utilized to identify a type among the three bacteria. To collect the data, the proposed sensor needs an impedance measurement system, as shown in Fig. 1, where an Agilent semiconductor analyzer B5100 is utilized for current-voltage (I-V) characteristics. As shown in Fig. 3a and b, the biosensor was loaded into the probe station, and the probes were connected to the terminals at ambient conditions. Bacteria cells were casted on the sensor with the help of a micro-pipette tip (1 μL), as shown in Fig. 3c, and a voltage sweep of ±2.5 V was applied across the terminals of the biosensor to characterize the I-V curve. As a result, the response time of the sensor was analyzed by injecting the bacteria sample and measuring the impedance with respect to time under a fixed 2.5 V. Initially, the sensor showed variation in the impedance. However, after 8 min of the sample injection, the impedance became stable for all three types of pathogens. The impedance became stable due to the interaction of bacteria with electrodes by making conductive paths between the fingers of the sensor with the help of AgNWs. For example, the *E. colisalmonella* bacteria cell was measured against a concentration of 10^4^–107 CFU/mL, and the measured impedance went to a steady state after 8 min, as shown in Fig. 3d.

**Figure 4:**
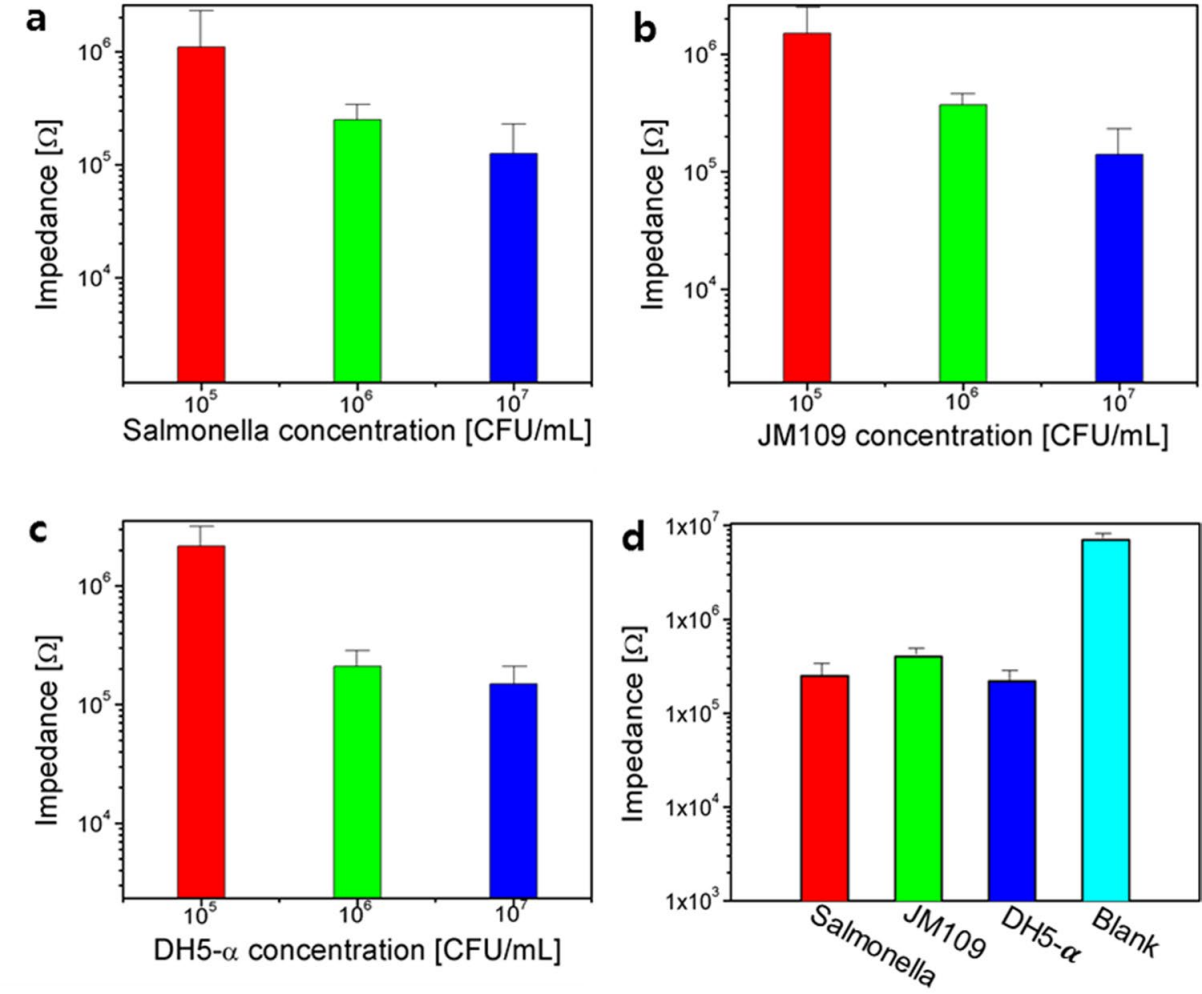
Impedance of the sensor against bacteria concentration: (**a**) DH5-α, (**b**) JM109, (**c**), salmonella, and (**d**) combined impedance response of the sensor at 10^6^ CFU/mL.

To classify three types of pathogens, *E. coli* JM 109 and DH5-α, and *Salmonella*, 40 samples of each bacteria type were measured at the same concentration of 10^6^ CFU at an ambient temperature. The temperature had no effect on the measurement since the measurement took place immediately after injecting the bacteria sample onto the sensor. Hence, the movement of the bacteria was not affected by the temperature variations. Each measurement contained 251 data points that were represented by a vector, $x=({x}_{1},{x}_{2},\ldots ,{x}_{251})$. The voltage sweeping of ±2.5 V with 251 steps was applied, which resulted in 251 of current (I) values, and these values are represented by *x*_1_ up to *x*_251_ (See section 6 in supplementary information for the detailed descriptions.). Each component of the data vector represents the current (I) value; e.g., *x*_1_ and *x*_251_ denotes the 1^st^ and the last current values at −2.5 V and 2.5 V, respectively. Figure 4a indicates the measured impedance data against ±2.5 V sweep for the salmonella against concentrations of 10^5^, 10^6^, and 10^7^ CFU/mL, which can be seen that the impedance value inversely varies with the concentration of bacteria. These impedance relations are because the higher concentrated bacteria creates more electrical paths between the electrodes’ fingers compared to a lower concentration. For E. coli JM109 and DH5-**α** samples, a similar trend was also observed, as shown in Fig. 4b and c. Figure 4d shows the impedance comparison of three selected bacteria with the base solvent (blank), as well as the settled impedance values of the *E. coli* DH5-**α**, JM 109, and salmonella.

As shown in Fig. 3d, the measured impedance before 8 min was not stable and the classification of the bacteria type was difficult. After 8 min, the impedance became stable. However, the difference among the impedance of three types of bacteria has a small value, which is difficult to detect with a conventional ohmmeter. To make a reliable classification of pathogens using both linear and nonlinear classification methods, Matlab R2015a was utilized. Unique fingerprints of the sample, including the first and second derivative, power, and energy, were extracted as described in the discussion section. These features were used in pattern recognition methods to classify microbes by using MLE, LDA, and BPNN, as detailed below:

### MLE

**Figure 5:**
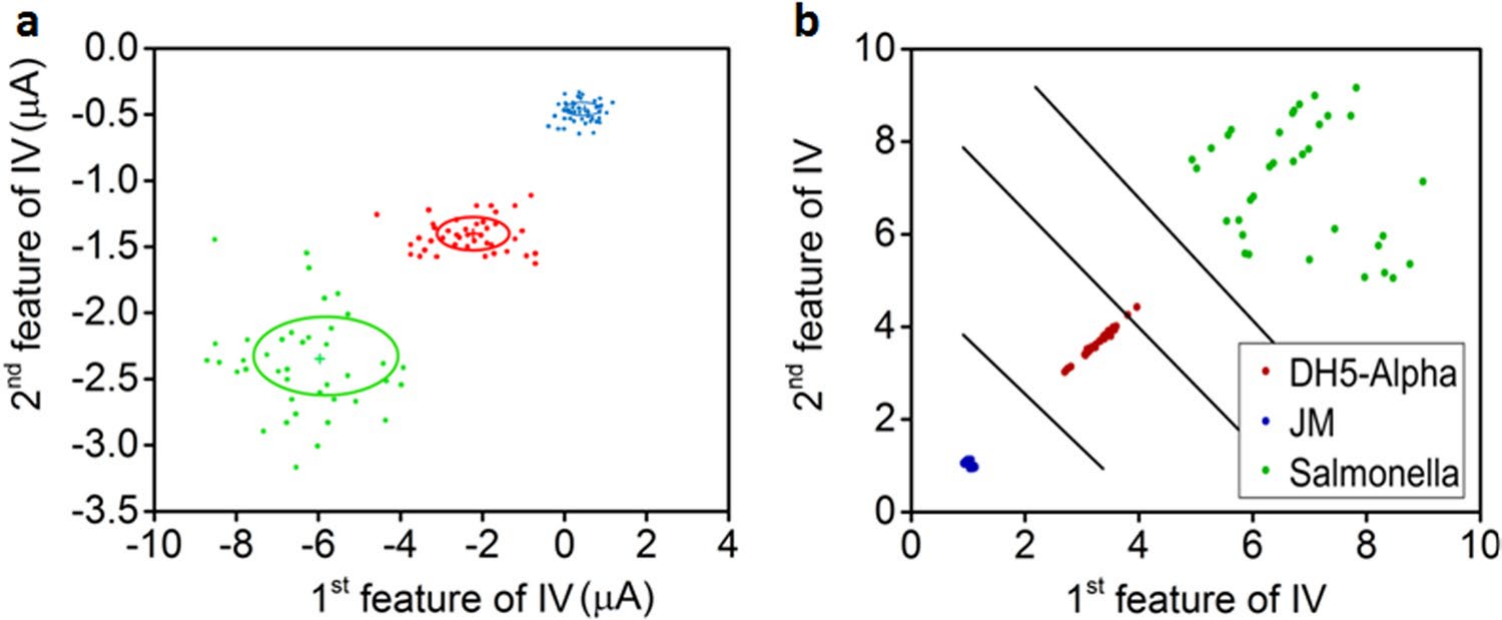
(**a**) Prediction of samples of three types of bacteria using MLE model (*E. coli* DH5-α, JM 109, and Salmonella are red, blue, and green color, respectively). (**b**) Cluster plot with LDA model for three types of bacteria.

As shown in Fig. 4d, each bacteria type has its own individual impedance variation for the fingerprint. By using a simple visual investigation of the feature differences or I-V response, all three types were rapidly classified by using the classical statistical MLE method^36–38^. Only two significant features (125^th^ and 126^th^) among the data points {*x*_1 …_ *x*_251_} were selected from the current data vectors. Forty observations were collected for each class, and the training samples among them were randomly selected; 70% of the data set were used to calculate the mean and covariance of each class. The ML classifier used this information of the mean and covariance to find the best estimate of the given testing sample. Figure 5a shows the result of the ML classifier with a suitable color illustrating different classes with a distinguished center, and a circle indicating the mean and covariance of each class, which are clearly separable. The accuracy analysis of the ML classifier was carried out by a square confusion matrix by comparing the identified class sample with the reference class. The confusion matrix’s rows and columns were equal to the number of classes and the diagonal entries represented the correct samples of each class while the remaining entries were misclassified. The achieved overall accuracy of the ML classifier was 100%, indicating correct classification of all the testing samples as compared to the reference samples. This high accuracy of MLE was due to the usage of good features, which were extracted from the measured data, as shown in Figures S11 and S12 in the supplementary information.

### LDA

Fig. 5b graphically illustrates the result of the LDA on the given data set. The measured data set includes the 1^st^ and 2^nd^ derivatives of the I-V data. In the scatter plot shown in Fig. 5b, the x-axis and y-axis represent the 1^st^ and 2^nd^ features of the 1-V data, respectively. These are the 120^th^ and 121^st^ current values, which are called features of each class. In the scatter plot, three distinguished cluster trends show *Salmonella* and *E. coli* JM 109 and DH5-α. The *Salmonella* had more variance as compared to *E. coli* JM 109 and DH5-α. The *Salmonella* also showed 47% variance, while *E. coli* JM 109 and DH5-α showed 34% and 19% variance, respectively, summing to a total of 100% data variance. As the analysis of scattering plot shows in Fig. 5b, there was a clear separation between the three bacterial groups. All the testing samples were well clustered along the two hyper planes with the exception of a few samples among these bacterial groups at the edges of the LD’s planes. The achieved overall accuracy by LDA is 100%, indicating accurate discrimination of the bacterial types.

### BPNN

**Figure 6:**
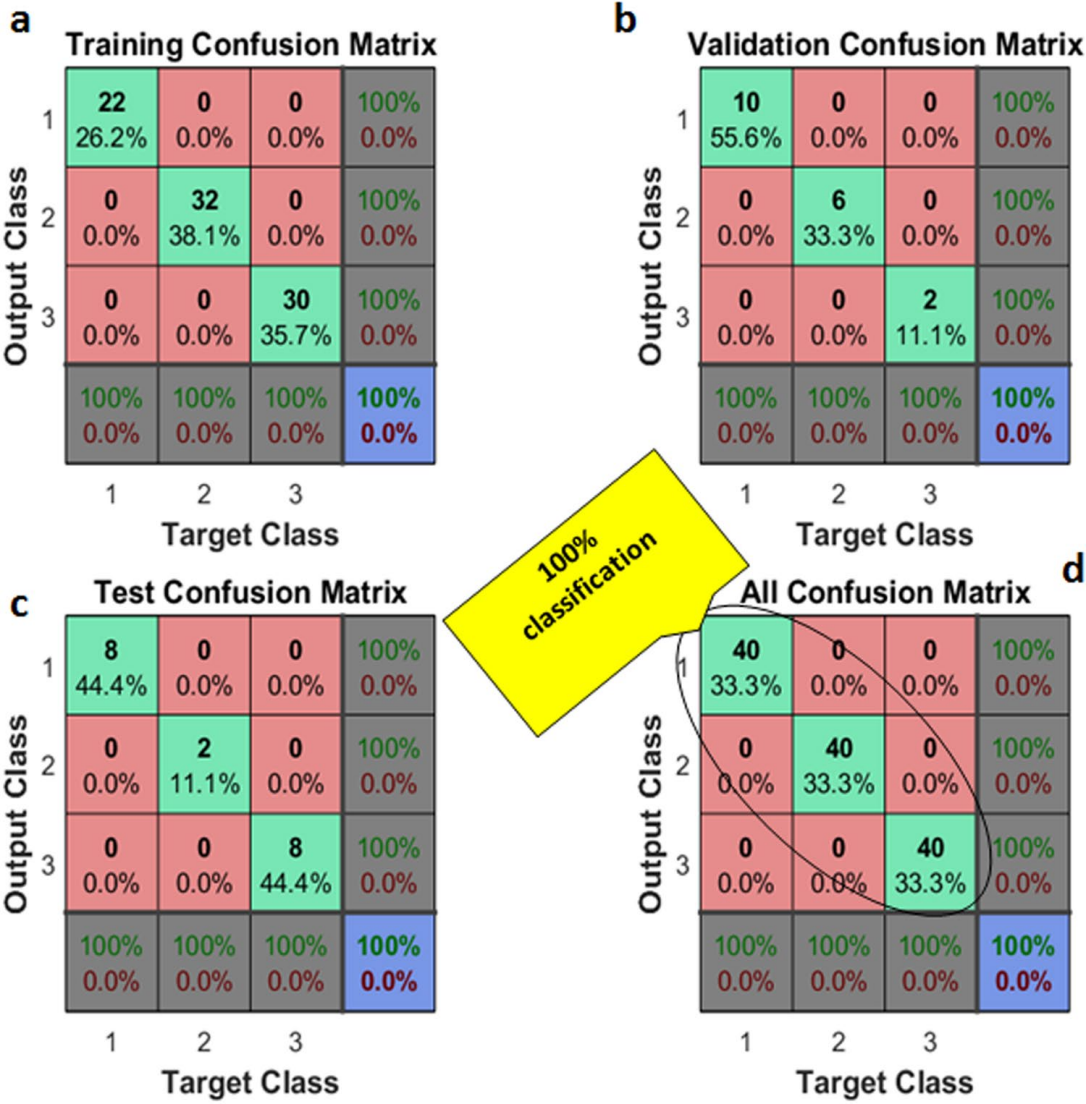
Classification of bacteria by using neural network (NN) with (**a**) 70% training, (**b**) 15% validation and (**c**) 15% test data. (**d**) All 40 samples of each bacteria type are correctly classified.

Previously, linear models were applied to the subjective problem, which produced precise classification results. Now, in contrast to the linear model, a nonlinear BPNN was applied to the given classification problem with a data set. The configuration of BPNN consisted of an input layer, a hidden layer, and an output layer. All of the features were used as an input with the target labels. The total number of nodes in the hidden layer and output layer were 10 and 3, respectively (See Figure S13 in supplementary information.). Other BPNN model parameters were selected as follows: the data were split into random training, testing, and validation sets, “tanh” was used as the activation function, and error gradients were found by the conjugate gradient. The objective function was cross entropy, while the learning rate and maximum iteration length were set to be 0.1 and 1000, respectively. Out of a total of 120 data vectors (100% data), 84 vectors (70% data) were used as training, while the remaining 36 vectors (30%) were divided equally between the testing and validation data sets, as shown by the confusion matrices in Fig. 6a,b and c, respectively. The accuracy analysis of BPNN was carried out by a square confusion matrix. All the samples were sorted in a diagonal position within a confusion matrix that has 131 interactions. The BPNN achieved 100% accuracy in training, testing, and cross validation, and all bacteria samples were correctly classified, as shown in Fig. 6d. (See supplementary section 6 for more detail.).

## Discussion

As shown in Fig. 4d, the resistance of three types of bacteria at 10^6^ CFU/mL was slightly different for each type because of their chemical properties, and these impedances made them distinguishable from each other. When the same types of bacteria samples were tested, there was also nominal resistance variation for each sample as represented by the error bar. These errors were due to the variation in the sample size, the position of the sample on the sensor’s surface, and the concentration of bacteria in each sample. Since the space between the fingers of the inter-digital electrode has an impact on the sensitivity of the sensor, it is important to find an appropriate finger space for the inter-digital electrode. For example, when the finger separation is l00 µm, there can be short-circuiting because of AgNWs, which makes it difficult to control the proper density during the EHD spray deposition. For this reason, from using the measured resistance variations from this finger spacing, the classification of the three types of pathogens becomes difficult. Although a small amount of each type of bacteria is measured, it has almost the same resistance due to interconnections between the fingers.

On the other hand, the sensors that have a larger finger spacing over 200 µm also have great impact on the sensitivity of the sensor. To make the connections in between the fingers for large spacing, a very high concentration of bacteria is needed because there needs to be a connection of at least 40–100 bacteria in series. In the large spacing case, some bacteria were exceptionally connected in the series to create a path between the fingers, and the resistance of the sensor did not change enough to detect the pathogens. Hence, a large spacing over 200 µm between the fingers reduces the sensitivity and cannot detect the bacteria efficiently. In this paper, we have experimentally used 200 µm finger spacing for three types of pathogens, which significantly detects the bacteria with good sensitivity (See section 1 in supplementary information for the best finger space).

AgNWs also affect the performance of the proposed biosensor to detect random scattered bacteria. This is because a long chain of bacteria between the fingers is needed in order to complete the connected circuit without AgNWs. Hence, the AgNWs enable the sensor to detect easily the random scattered bacteria in a short time without making a chain between the fingers, which requires a very long time. To detect three types of pathogens, the density of AgNWs is important. For example, there is a short circuit between fingers when the AgNWs are deposited with high density, and a low density reduces the sensitivity. We experimentally selected the density of AgNWs as 30 × 10^3^/mm^2^ for maximum sensitivity (See section 2 in supplementary information for AgNWs concentration.).

The sensor with 200 µm spacing and a medium concentration of 30 × 10^3^/mm^2^ AgNWs can detect all concentrations of bacteria. However, a high concentration of 10^7^ CFU/mL as well as a low concentration of 10^5^ CFU/mL for the bacteria colony had a minimum effect on the performance of the proposed biosensor, but 10^6^ showed good sensitivity. Hence, a bacteria concentration of 10^6^ CFU/mL was used, which partially changed the impedance of the sensor while the machine learning algorithms efficiently classified the bacteria categories. The reference resistance was only due to a culture liquid medium without bacteria, and this reference resistance remained constant over time. When the homogeneous bacteria dilutions were casted over the sensor at ambient conditions, the bacteria moved down to the surface of the sensor through the culture medium and attached with AgNWs, which decreased the resistance between the fingers of the inter-digital electrodes. This process can take some time, which is called the settling time of the sensor, where the impedance of the sensor is not stable during this period. After the settling time, the bacteria on the surface of the proposed biosensor becomes almost stable. Hence, it gives a constant resistance. Each type of bacteria has a different size, shape, and biochemical composition. Hence, these are the main factors that can lead to a slight variation in terms of the electrical resistance against each bacteria colony.

For testing purposes, we cultured bacteria with these characteristics: *Salmonella* cells had diameters between approximately 0.7 and 1.5 µm and lengths from 2 to 5 µm, which were rod-shaped motile bacteria that possessed flagella. The *E. coli strains* JM 109 and DH5-α were typically rod-shaped and had a diameter from about 0.25 to 1.0 µm and a length of about 2.0 µm. *S. typhimurium* and *E. coli* showed a high degree of similarity on their genome contents as well as the overall shape described above. Both microorganisms were Gram-negative facultative anaerobe bacteria and part of the family of Enterobacteriaceae. Hence, they were closely related bacteria. However, two bacteria had different patterns of carbon utilization^39^ and lipid composition^36^. *E. colistrains* JM 109 and DH5-α were very similar strains, except for F’episome in *E. coli* JM 109. There were also some additional differences in genotype between the *E. coli strains* JM 109 and DH5-α.

By utilizing the cultured bacteria, the output impedance of the sensor was measured and normalization was done to make it consistent for the feature extraction. The normalization was done by dividing each measurement with a maximum value among the samples. Based on power, energy, and slop, the features were extracted from the collected data. These variables were used for linear analysis, and there were clusters of each bacteria type, as shown in Fig. 5. There was partial overlapping between the measured variables. However, the machine learning algorithms were used to get a good performance in terms of classifying different bacteria categories. Linear MLE, LDA, and non-linear BPNN tools were studied for classification, which clearly separated each bacteria type by classifying 100%. Hence, it was feasible to use these algorithms to classify these bacteria categories.

In conclusion, a new type of biosensor based on the resistance variation of a rapid detection of bacteria was proposed. The detection time was less than 10 min and the per unit cost was less than 1 USD/unit. To verify the biosensor, it was applied to classify different types of pathogens through pattern recognition methods by using the measured data from the sensor. The biosensor based on comb type highly conductive silver electrodes was fabricated through printed inkjet technology at ambient conditions and standard atmospheric pressure. To increase the detection for random scattered samples, AgNWs were sprayed over the printed biosensor by the EHD technique. The quantitative concentration of microbes 10^6^ CFU/ml was used for the identification process. The current of the proposed biosensor varied and reached up to few micro amperes for different bacteria types when a voltage of −2.5 to 2.5 V was applied across its terminal. The Agilent B1500A semiconductor device analyzer and the probe station were utilized to measure the I-V characteristics of three popular bacterial types: *E. coli* JM 109 and DH5-α, and *Salmonella*. Unique fingerprints or features (I-V curve, slop, 2^nd^ derivative, and energy) were extracted from the measured I-V characteristics from the proposed electronic biosensor. Linear MLE, LDA, and nonlinear BPNN pattern recognition methods were used to classify three food pathogens. All the samples were correctly classified with 100% accuracy by using the MLE, LDA, and BPNN pattern recognition methods. The overall results show the proposed scheme is simple, fast, accurate, and economical. Hence, it can be utilized in the food industry as an early detection and classification method.

## Methods

### Sensor

#### Design

The proposed biosensor consists of silver inter-digital electrodes decorated with silver nanowires on a plastic, 100 µm thick PET substrate. The dimension of the biosensor was experimentally selected with a finger width of 100 µm and fingers spacing of 200 µm, as shown in Fig. 2a, and an AgNWs density of 30 × 10^3^/mm^2^ (See the detailed description in supporting information about selecting the best sensor.).

#### Materials

The electrodes of the sensor were made of silver nano particle ink that was prepared as follows: First, silver nanoparticles (purchased from ANP South Korea) 55 wt% were diluted in a 10 mL ethylene glycol solvent and mixed for 2 hours by a magnetic stirrer. Then, there was bath sonication for 25 min. The Ag ink had a viscosity of 11.3 mPa.s (by using the Viscometer VM-10A system), a surface tension of 36.8 mNm (analyzed with surface electro optics), and a specific gravity of 1.66 gm/mL. The second material used in fabricating the sensor was silver nanowires (AgNWs) that were purchased from Sigma Aldrich in South Korea. The average length and diameter of the silver nanowires were in the µm and nm range, respectively.

#### Two steps fabrication

The sensor was fabricated in a two step process. First, the comb type electrodes of the biosensor were designed in ACE-3000 and fabricated by a DMP-3000 material inkjet printer. In the second step, the silver nanowires were sprayed over the surface of the sensor using the EHD technique. The electrode file designed in ACE-3000 was converted into a compatible file format for the DMP-3000 material printer, loaded into DMP-3000, and silver ink (3 mL) was filled in a cartridge that contains 16 nozzles. The substrate was treated with a UV ozone cleaner for 30 sec and then loaded into the inkjet printer. The printing process and setup conditions such as drop spacing, drop velocity, heating temperature, number of layers to be printed, and number of nozzles used in printing and height of the substrate were selected in the Dimatix Drop Manager (DDM). Before the biosensor was printed using the Fuji Film Dimatix DMP-3000 printer, the software design was exported to ACE 3000 software in a Gerber file format that contains all the geometrical dimensions of the electrodes design. The converted file in the DMP-3000 was used for printing the electrode from a material inkjet printer.

**Figure 7:**
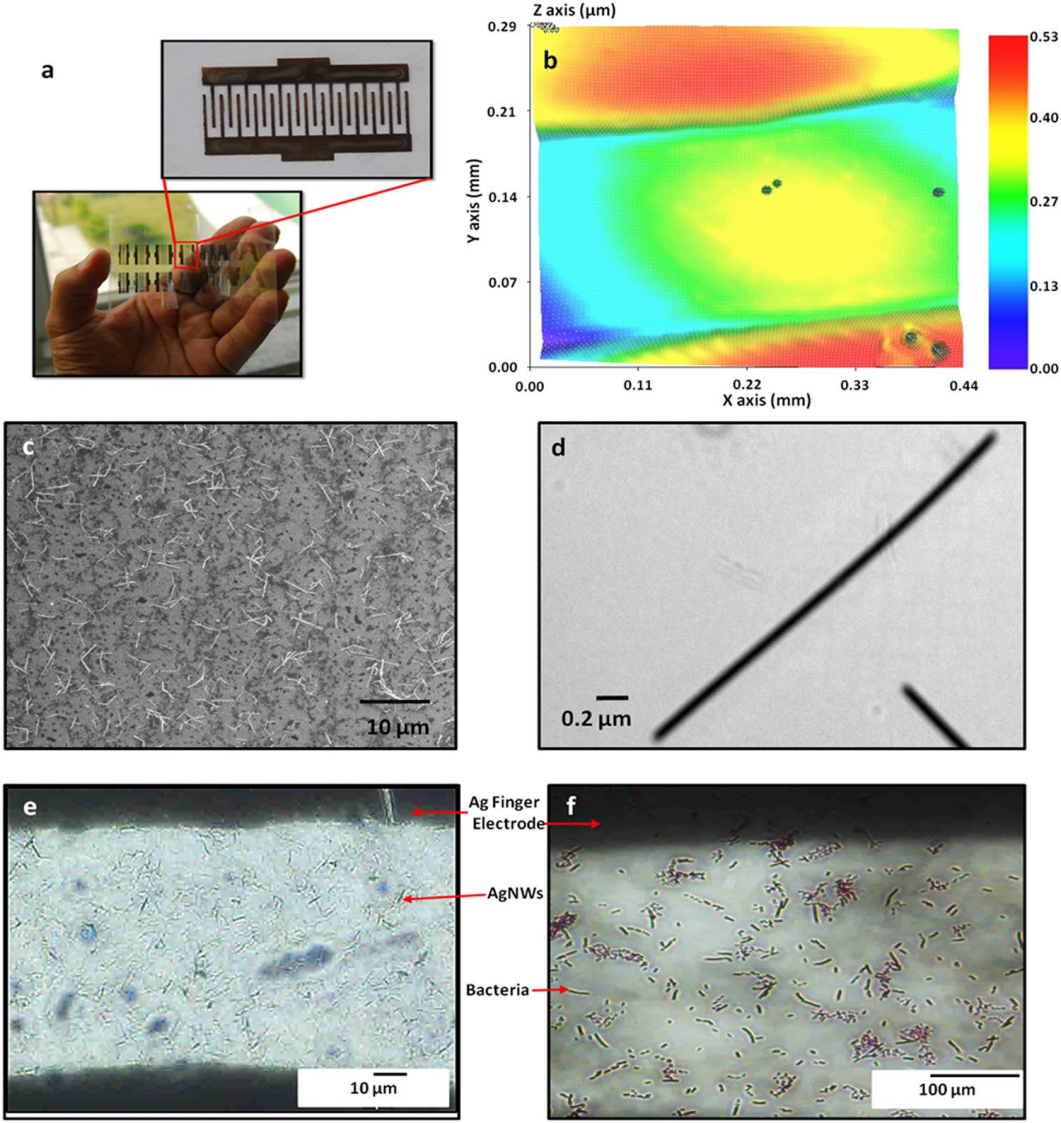
(**a**) Photograph of the fabricated bacteria sensors on a PET substrate, the zoomed image is shown in the inset. (**b**) 3D nano profile image of the Ag bare electrodes. (**c**) SEM image of the Ag nanowires between the electrodes. (**d**) TEM image of the AgNWs spread over the electrodes. (**e**) Optical microscope images of AgNWs. (**f**) Optical microscope images of bacteria over the electrodes of sensor.

**Figure 8:**
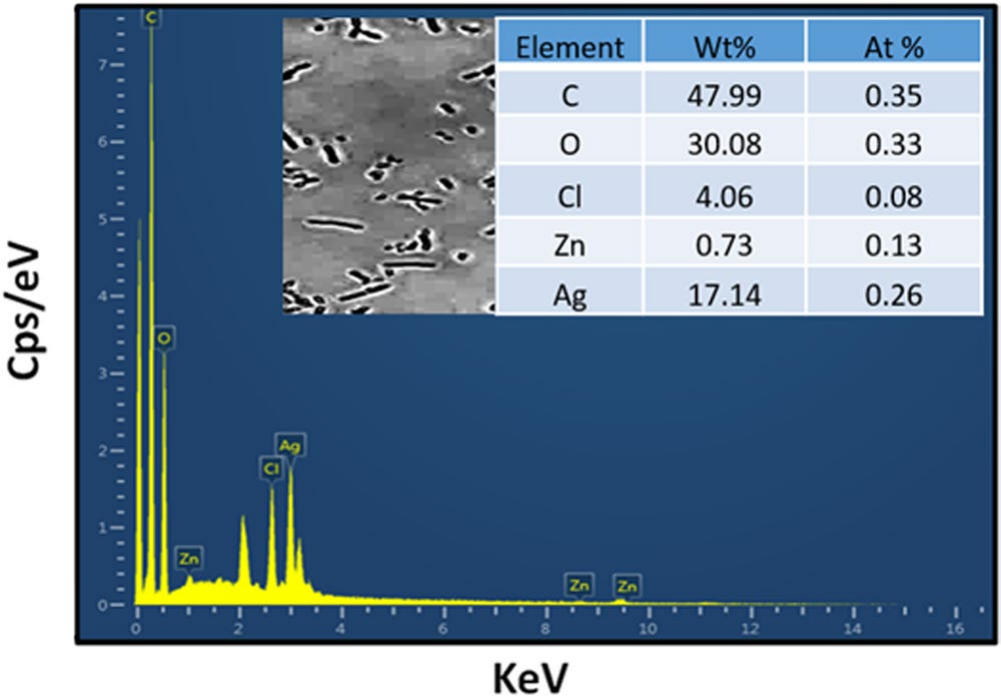
EDS analysis of the sensor between two finger electrodes.

We deposited two layers of silver ink on the PET substrate to increase the electrical conductivity as well as to achieve continuous conductive patterns (See section 4 in supplementary information for more details). After depositing the silver ink for the electrodes, the silver ink was cured at 100 °C for 30 min. The fabricated biosensor’s electrode is shown in Fig. 7a. NV-2000 (Universal) non-contact surface profiler with a nano level accuracy was used for surface morphology measurements in a phase shifting interferometry (PSI) mode. As the 3D profile of Ag electrodes is shown in Fig. 7b covering two fingers and space between them, it can be seen that the electrodes deposited through the inkjet printer are uniform with a thickness of almost 400 nm. The bacteria detection was difficult as the spacing between the fingers was 200 µm, while the size of the bacteria was less than 4 µm. Hence, we used AgNWs to reduce the spacing between the electrodes, which helped in detecting a lesser amount of bacteria, easily. The AgNWs were decorated on the electrodes through the EHD system with an average density of 30 × 10^3^/mm^2^ (See section 5 in supplementary information about AgNWs deposition using EHD.). The average length of the AgNWs varied between 2 to 5 µm and the length of bacteria was 0.2 to 5 µm, as shown in Figs 7d and 8a,b, respectively. In the case of electrodes without AgNWs, a chain of 40 to 100 bacteria was needed to make a finger to finger electrical connection, which can cause changes in impedance. This detection is rarely possible in a high bacteria concentration. Hence, the sensitivity of the sensor is very low and it cannot be used for low and medium concentrations of bacteria as the detection of these concentrations are important in the food industry (See section 3 in supplementary information about bacteria concentration).

After decorating AgNWs on the electrodes, the average spacing between the two AgNWs is the same as the length of the bacteria where a lesser amount of bacteria can make connections from finger to finger and enable the sensor to detect a lesser amount of bacteria. The scanning electron microscopy (SEM) image (taken through Jeol JSM-7600F) of decorated AgNWs on the electrodes is shown in Fig. 7c. Because of the non-uniform deposition of AgNWs, they are making chunks, but they are still not making a short circuit between fingers. The surface morphology of the AgNWs was also studied using transmission electron microscopy (TEM) as the corresponding image given in Fig. 7d. It shows the diameters of nanowires in nm range and lengths of tens of micrometer. The optical microscope images of AgNWs and bacteria between the inter-digital finger electrodes are shown in Fig. 7e and f. The spacing between the bacteria is in micro meter that can be occupied with an average concentration of AgNWs (See section 2 in supplementary information about AgNWs concentration.). The sensor was analyzed with energy dispersive spectroscopy (EDS) on order to confirm the elements present on the surface of the sensor. During the EDS analysis through SEM, the sensor surface contain AgNWs and *E.Coli* bacteria. The EDS spectrum and tabulated results revealed that Ag, C, Cl, and Zn are the main elements present with C being the most abundant in the selected field as shown in Fig. 8. The Ag peaks originate from the AgNWs, whereas C peaks originate from the PET substrate, Cl and Zn are present due to the E-Coli bacteria and its solvent.

### Bacterial Colony Preparation

In this paper, the *E. coli strains* JM 109 and DH5-α purchased from Promega Corporation and the *Salmonella* strain kindly provided by Prof. Tatsuya Unno in Jeju National University were used. The three bacteria strains were cultured on a Luria-Bertani (LB) solid medium at 37 °C for 16 hours to obtain a single colony. The single colony of each bacteria cell was picked up and cultured in an LB liquid medium at 37 °C for 16 hrs with shaking at 180 rpm. After the culture, the bacteria cells were collected by centrifugation at 3000 rpm for 5 min and the supernatant was removed. The collected bacteria cells were re-suspended in 1 mL of deionized water (DW) and the cell density was checked using a spectrophotometer. The re-suspended cells were diluted with DW to adjust the cell density from 10^4^ to 10^7^ colony-forming units CFU/mL.

### Measurement

In measurement, we loaded the sensor in the Agilent probe station. Then, we casted the bacteria over the sensor and measured the electrical characteristics.

#### Casting the bacteria over sensor

The sensor was loaded on the adjustable stage of the Agilent probe station, as shown in Fig. 3a, and probes were connected across the sensor. The prepared solution of each type of bacteria was casted over the sensor by using a 1 µL micropipette tip. At each time, a new sensor was used to measure the data of each bacteria sample. The zoomed image of the sensor can be seen in Fig. 3c. The optical microscope image of the sensor is shown in Fig. 7f, which clearly shows that the bacteria are present over the surface of the sensor.

#### Data Acquisition System

Each bacteria type was uniformly casted over the surface of the sensor to take a measurement by using the Agilent probe station. The resistance of the sample was measured across the terminal of the sensor. The sample data values were measured after 8 min of the sample injection on the sensor. A total of 120 measurements were taken from 40 samples of each type of bacteria.

### Classification

#### Feature extraction

The data set contained forty voltage and current vectors of each bacteria type. The sample impedance was measured from the voltage and current values for the voltage sweep of ±2.5 V. The characteristic variable of the current has scalar values, which has an important role in recognizing the bacteria type. This is because it has different characteristics for each bacteria type, which helps to classify the bacteria. We also extracted the first and second derivative of the current vector as feature vectors (See sub section 6.3 in supplementary information for more detail). Similarly, the characteristic variation of power, which is the scaled version of the current, is an important feature variable for classifying bacteria categories. Therefore, the current, impedance, current vector first and second derivative, power, and energy were extracted as feature vectors for classification. The obtained features, as shown in Figure S11, make a good classification of pathogens more easily, quickly, and accurately.

#### Algorithms

Both the linear as well as nonlinear machine learning algorithms were used to discriminate bacteria types. In linear algorithms, we selected the maximum likelihood (ML) and linear discriminant analysis (LDA) methods, as they are widely accepted as standard approaches and are simple and easy to implement. The MLE classifier is a statistically consistent approach for estimation and it becomes a minimum variance unbiased estimator as the size of the data set increases. This means the estimator has the minimum variance and the narrowest confidence interval of all estimators for that type. However, the computational cost of MLE is high and takes a longer time to estimate the data points as the data size increases. For this reason, LDA is an attractive linear classifier because it has design criteria based on maximizing class separability. For the nonlinear machine learning algorithms, we used BPNN, which is a popular, powerful, self-adaptive, and flexible algorithm. It also has the capability of capturing nonlinear and complex underlying characteristics of any physical process with a high accuracy. However, its convergence is slow, but guaranteed. BPNN is a black box learning approach and the user cannot interpret the relationship between input and output to deal with uncertainties. It is also complex as compared to MLE and LDA due to it being nonlinear and consisting of a layered configuration, where the error back propagates and then the weights in the layers update to minimize the error. The extracted feature variables were provided to LDA, MLE, and BPNN, and the performance of all these algorithms were evaluated to discriminate bacteria classes. The data analysis process that used machine learning algorithms was carried out in Matlab R2015a (Mathworks) on MS Windows 7.

## Electronic supplementary material

Supplementary information
