# Supplementary material for: Disposable all-printed electronic biosensor for instantaneous detection and classification of pathogens
Source: Sci Rep. 2018 Apr 12;8:5920. doi: 10.1038/s41598-018-24208-2 (PMC5897556; doi:10.1038/s41598-018-24208-2)

**Supplementary Information: Disposable all-printed electronic biosensor for instantaneous detection and classification of pathogens**

**Shawkat Ali^1,4^, Arshad Hassan^1,4^, Gul Hassan^1^, Chang-Ho Eun^2^, Jinho Bae^1^*,**

**Chong Hyun Lee^1^, and In-Jung Kim^2,3^**

*^1^Department of Ocean System Engineering, Jeju National University, 102 Jejudaehakro, Jeju 63243, South Korea*

*^2^Subtropical/tropical Organism Gene Bank, Jeju National University, Jeju, 63243, Republic of Korea*

*^3^Faculty of Biotechnology, College of Applied Life Sciences, Jeju National University, 102 Jejudaehakro, Jeju 63243, South Korea*

*^4^Department of Electrical Engineering, National University of Computer and Emerging Sciences FAST, H 11/4, Islamabad 44000, Pakistan*

^*^Present address: Department of Ocean System Engineering, College of Ocean Science, Jeju National University, 102 Jejudaehakro, Jeju-si, Jeju Special Self-Governing Province 63243, Republic of Korea

^*^E-mail: [baejh@jejunu.ac.kr](mailto:baejh@jejunu.ac.kr), (Phone) 82-64-754-3483, (Fax) 82-64-755-6130

**1. Finger spacing**


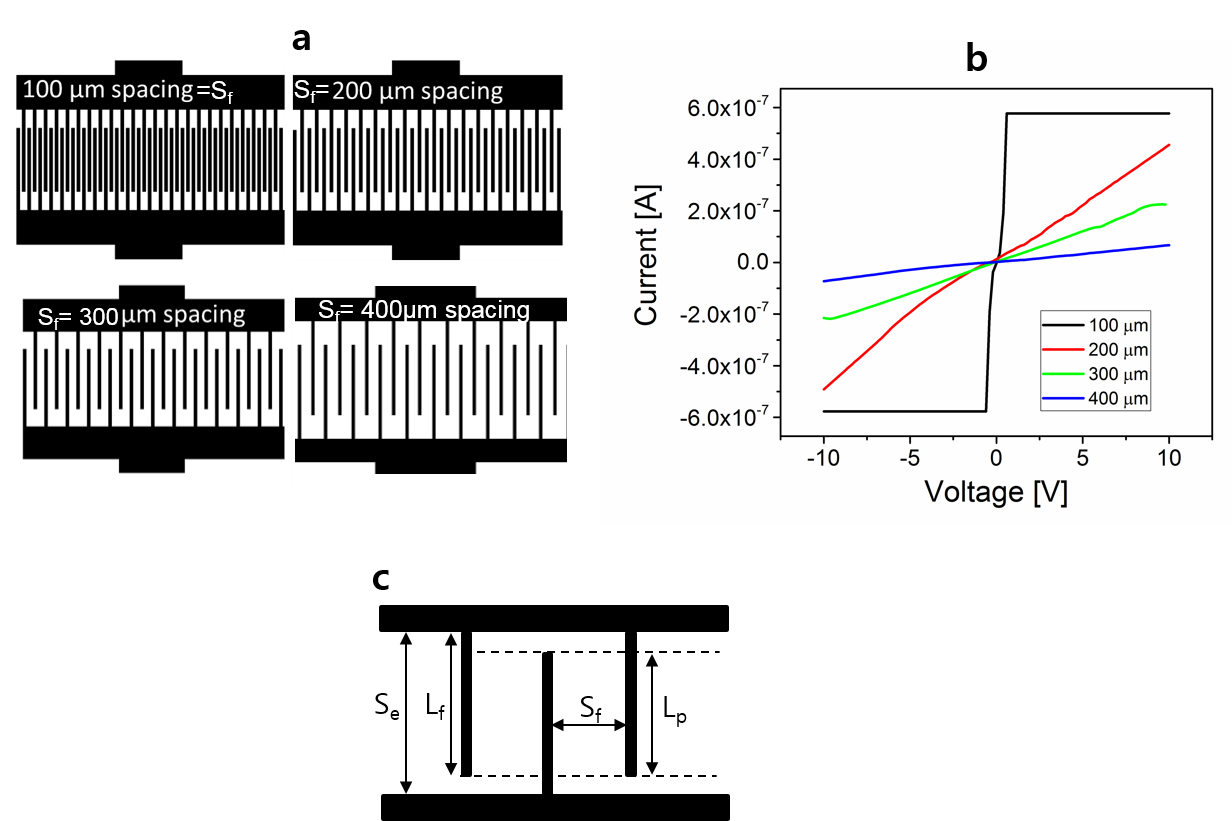


Figure S1. (a) Four types of inter-digital electrodes with, S_f_ = 100, 200, 300, and 400 µm spacing.(b) Electrical response of the sensor at different fingers space size. (c) Schematic diagram of the sensor’s electrode.

Inter-digital patterns of 100, 200, 300, and 400 µm finger spacing (S_f_) were fabricated as shown in Figure S1a. All electrodes were decorated with AgNWs of density 30×10^3^/mm^2^. By using same bacteria sample (high concentration of bacteria 10^7^ CFU/ml), the sensor showed different behavior as shown in Figure S1b. The 100 µm finger spacing pattern electrode showed short circuit behavior as shown with black line because the spacing is small and fingers of electrodes get connected electrically due to AgNWs. The behavior of 200 µm spacing electrode is shown with red line in Figure S1b, which shows a linear relationship between current and voltage. This result shows that there is certain resistance of the sensor which is readable for the range of voltages and its sensitivity is high. By using 300 µm spacing pattern, the electrical behavior is linear however, the current is less as shown with green line in Figure S1b, and the sensitivity of the sensor is low as compared to the sensor with inter-digital finger spacing of 200 µm. By increasing the finger space to 400 µm, the electrical behavior is linear but the current is not prominent as shown with blue line in Figure S1b. The current value is directly proportional to the sensitivity of the sensor. Hence, the best value of finger spacing was experimentally found to be 200µm as the behavior is prominent and its sensitivity is a high.

The sensor parameters such as finger length (L_f_), finger spacing (S_f_), fingers parallel length (L_p_), and separation between the electrodes (S_e_) are playing vital role in the sensor response. Increasing the L_f_, it also increases the L_p_, hence parallel area between the fingers is increased and impedance of the sensor is decreased while the concentration and amount of bacteria is fixed. Increasing the S_f_, sensitivity of the sensor decreases at fixed concentration of bacteria and S_e_ controls the L_p_. This experiment is done with bacteria concentration of 10^7^ CFU/mL to analyze the effect of parameters of the electrodes. Optical images of the fabricated sensors are shown in Figures S2aand S2b. These figures show that the electrodes were properly fabricated without short circuiting the finger with each other.


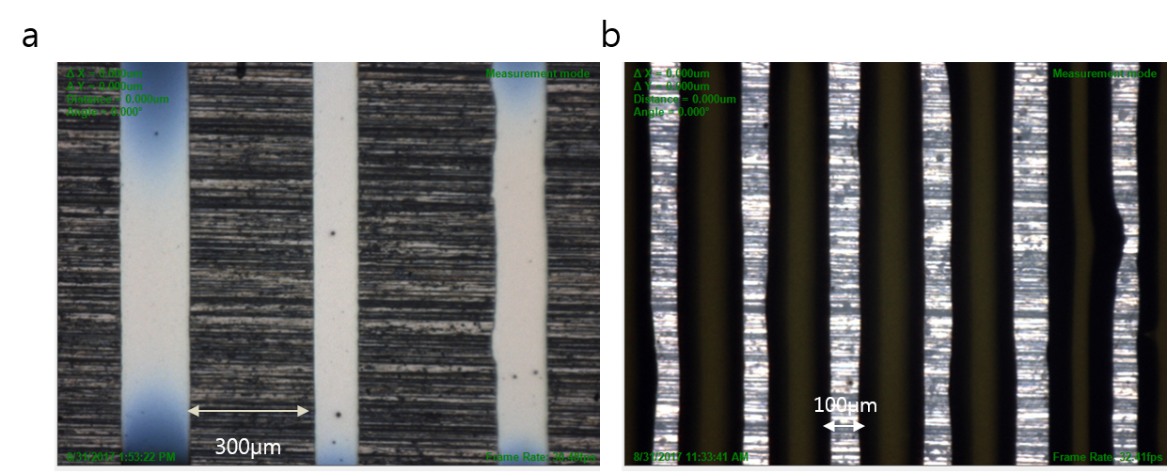


Figure S2. Inkjet printed electrodes with finger spacing of (a) S_f_ = 300µm and (b) S_f_ = 100µm.

**2. AgNWs concentration**

The sensor with finger spacing of 100µm when decorated with small amount of AgNWs (10x10^3^/mm^2^), it suffers due to short circuit as there is small gap between the fingers and we deposited AgNWs through EHD technique. In the above section, we selected the best finger space of 200 µm. To increase the detection of the bacterium, the concentration of AgNWs on the electrodes was studied, where we have deposited three concentration such as low, medium, and high concentration (10×10^3^/mm^2^, 30×10^3^/mm^2^, and 50×10^3^/mm^2^) of AgNWs, and the optical images are presented in Figures S3a-c. The low concentration of AgNWs with 200µm finger spacing only detects high concentration of bacteria and cannot applicable for low and medium concentration of bacteria. With medium concentration, it can detect all concentration of bacteria with significant margin in impedance change. The high concentration of AgNWs makes the inter-digital electrodes (fingers)short circuit and it has similar trend as with 100 µm finger spacing that is shown in Figure S3d.


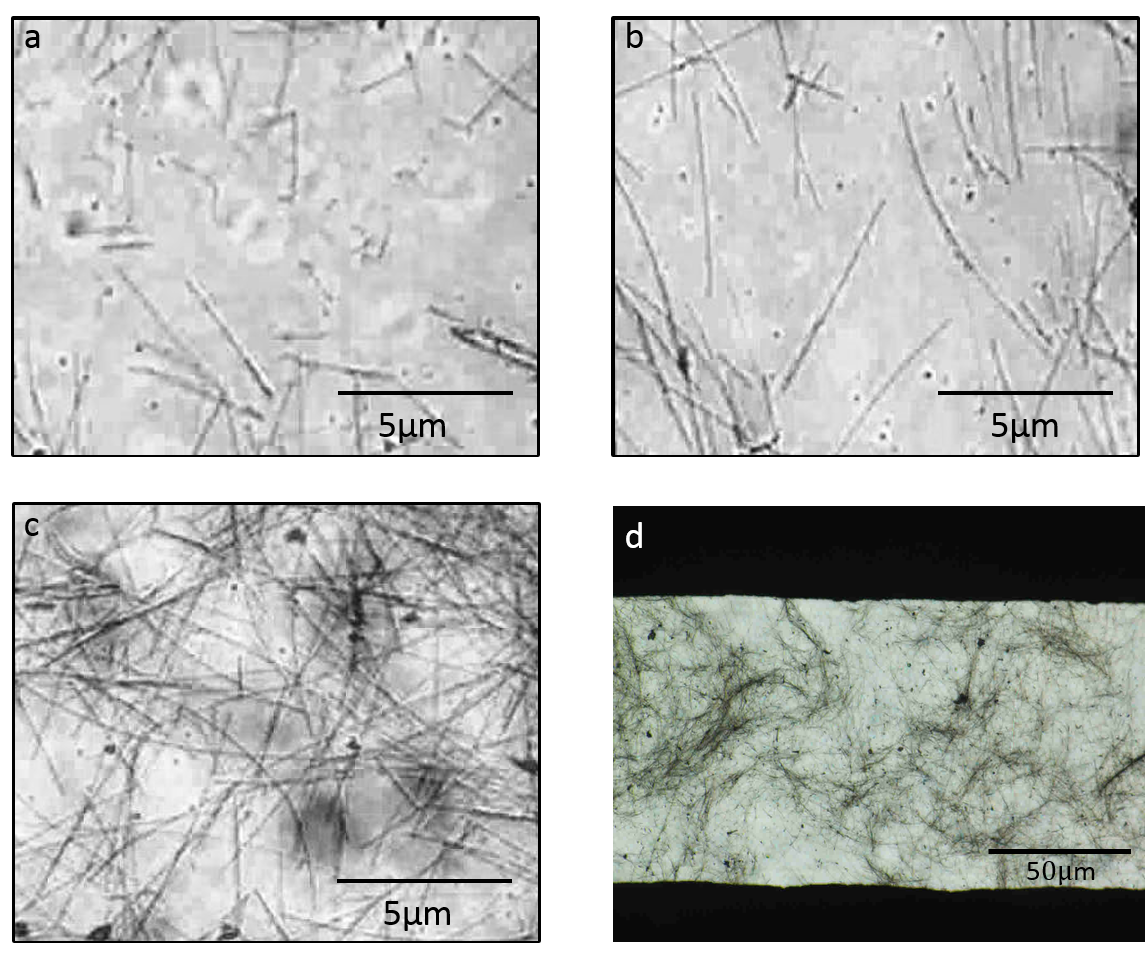


Figure S3. Zoomed image of sensor with (a) low, (b) medium and (c) high concentration of AgNWs. (d) Microscopic view of high concentration of AgNWs between inter-digital electrodes.

After that, we have tested 300 µm finger spacing sensor. With a low concentration of AgNWs, the sensor did not show response against all concentration of bacteria. While the medium concentration of AgNWs showed response only for the high concentration of bacteria. The sensor with a high concentration of AgNWs gives response for medium and a high concentration of bacteria. However, the sensitivity is very low as compared to previous case. Moreover, it cannot detect low concentration of bacteria. We also analyzed the sensor with the finger spacing of 400 µm against AgNWs concentration. The sensor showed open circuit behavior even a high concentration of AgNWs and bacteria is used, as there is large space between the fingers. From the above AgNWs analysis, we selected the medium concentration 30x10^3^/mm^2^ of AgNWs and 200 µm inter-digital spacing as it works for all concentration of bacteria.

**3. Bacteria concentration**

The bacteria concentration was analyzed through optical microscope. Three concentration levels such as low (10^5^ CFU/mL), medium (10^6^ CFU/mL), and high (10^7^ CFU/mL) were measured as shown in Figures S4a, b, and c. Since it is clear from these images when we consider low concentration of bacteria, we need large amount of AgNWs to connect them. Similarly, in high concentration of bacteria, we need small amount of AgNWs to make electrical connection. Another important concern is, if there is contamination in food or in other useful resources, an immediate response is required to detect the alarming situation, immediately.


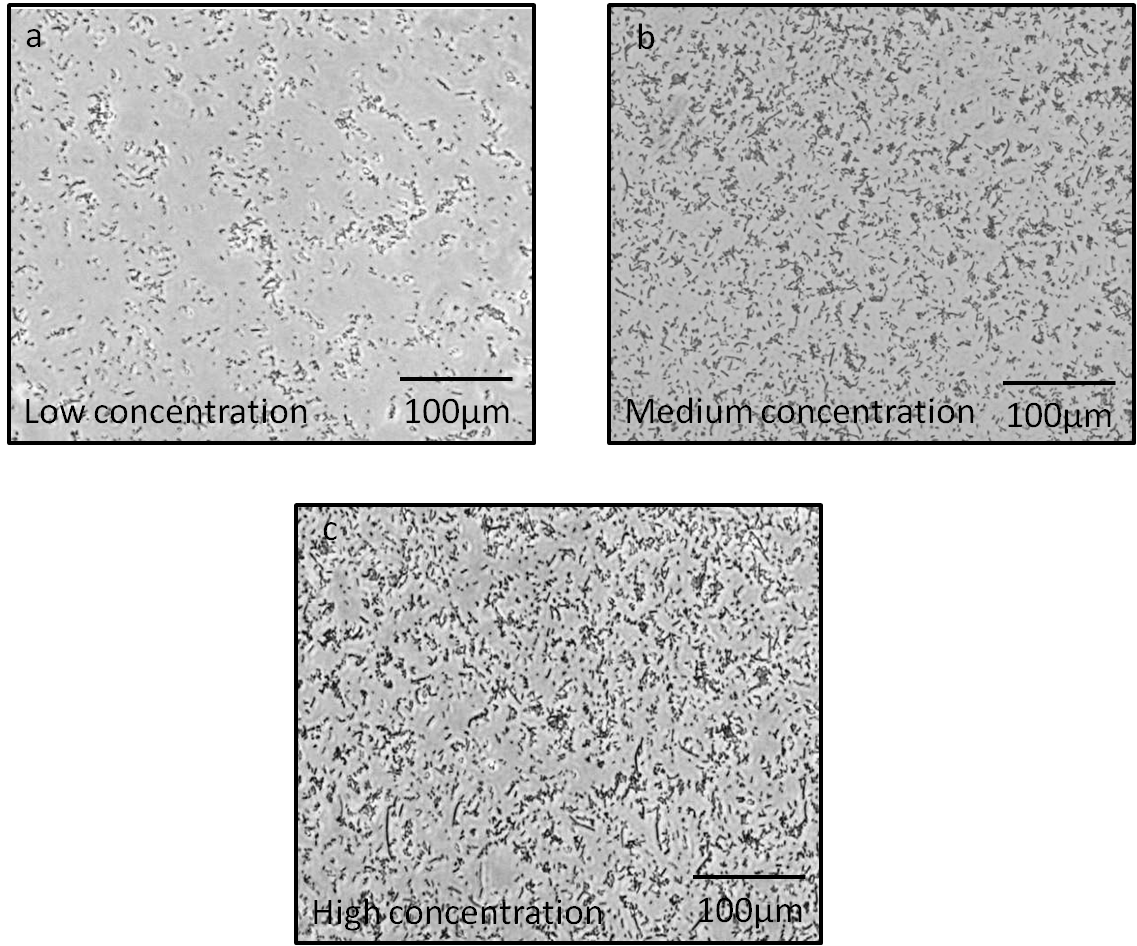


Figure S4. Optical microscopic images of bacteria concentrations (a) low, (b) medium, and (c) high.

**4. Electrodes fabrication with inkjet printer**

The inter-digital pattern of the proposed sensor is printed through Dimatix material inkjet printer (DMP-3000) as shown in Figure S5. This fabrication process is followed as; the PET substrate was UV treated for 30seconds,and then it was placed over the platen at 50°. The silver ink is loaded into the cartridge containing 16 nozzles with 10 pL. After all the printing parameters adjusted through DDM, it initiates and operates printing process. After this printing process is completed, the fabricated sensor was placed over the hot platen for 30 min to dry the Ag ink, and then AgNWs were deposited over the surface of the sensor through EHD technique.

**
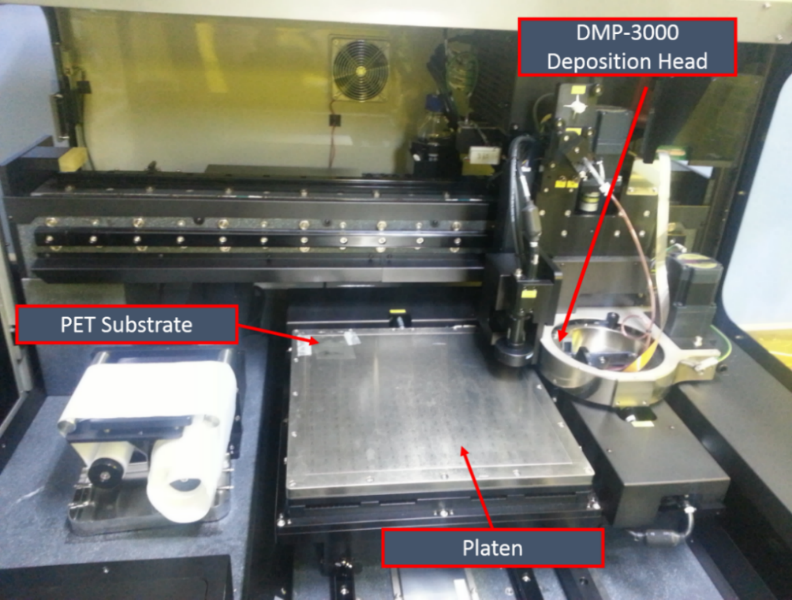
**

Figure S5. Dimatix material inkjet printer.

**5. AgNWs deposition**

AGNWs were deposited through EHD technique as the schematic diagram is shown in Figure S6. In the EHD printing method, the AgNWs ink was pumped through a nozzle at an appropriate flow rate with the positive potential at the nozzle and ground at the substrate. The induction of the surface charges on the pendent meniscus emerging at the nozzle outlet, results in an electric stress over the liquid surface. If the electric field and flow rate are in some operating range then this will overcome the surface tension stress over the liquid surface and results in deformation of the droplet at the orifice of the nozzle into a conical shape. Due to the tangential electric field acting on the surface of the liquid cone, a thin jet emanates at the cone apex which further breaks up into a number of small droplets under the effect of coulomb forces. Different spraying modes exists in EHD technology like dripping, micro-dripping, spindle, cone-jet, and multi-jet mode, but stable cone-jet is most important spraying mode because of the generation of the mono dispersed droplets with few micrometer diameters. To deposit the AgNWs using the EHD operation, our system was set up as; Nozzle has an internal diameter of 110 μm, which was placed at standoff distance of 12 cm and stage speed of 3mm/s. It was operated as flow rate of 150 μL/hr and applied voltage as 5.2 kV. The deposited sample was cured at 120 °C for 30 min.


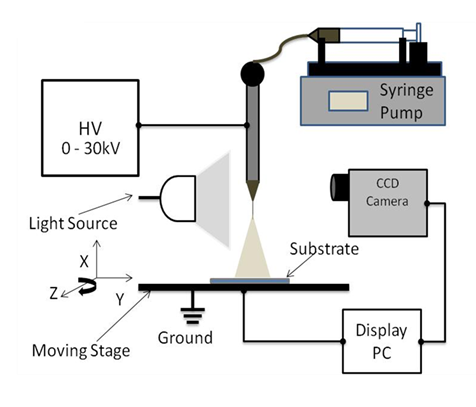


Figure S6. EHD schematic diagram.

**6. Data collection, feature extraction and classification using algorithms**

- 1. **Measurement setup**

The proposed sensor with bacteria is placed inside the probe station as shown in Figure. S7. Voltage is applied across the terminals and current values are recorded in a text file.


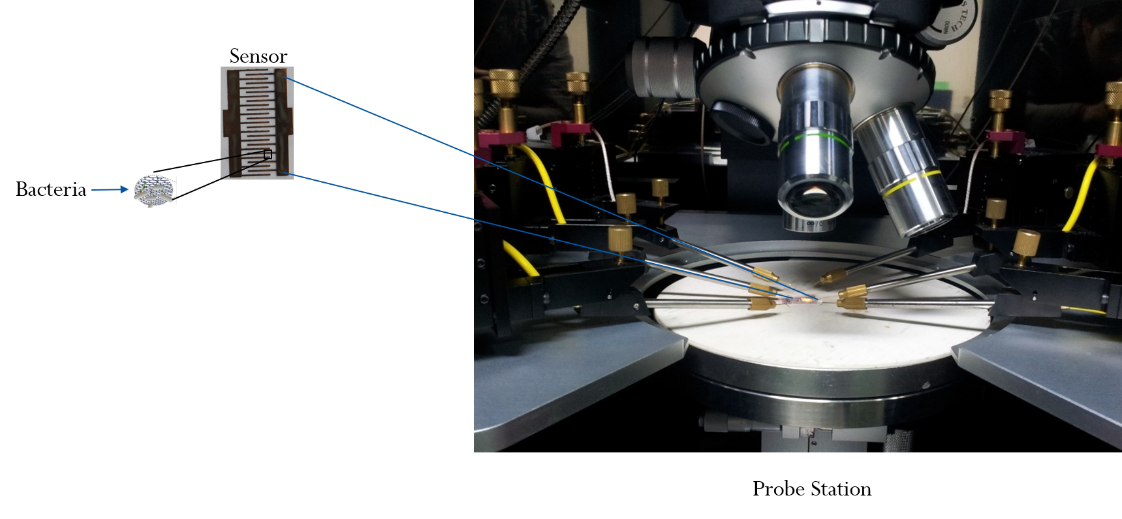


**Figure S7. Measurement setup.**

- 1. **Measurements**

In measurement, the voltage sweep of ±2.5V was applied across the terminals of sensor and the current value are recorded. The measured current for three bacteria types such as *E. Coli* DH5-α, JM109, and *Salmonella typhimurium* is shown in Figures S8a, 8b, and 8c, respectively. There are 251 current points against applied voltage range of ±2.5 V for each type of bacteria samples. We have used 10^6^ CFU/mL concentration of each bacteria sample in measurements. In our measurement, there is current at zero voltage as shown in Figure S8 due to capacitive effect of the inter-digital electrodes. Fingers of the electrode are in parallel with each other and create capacitance.


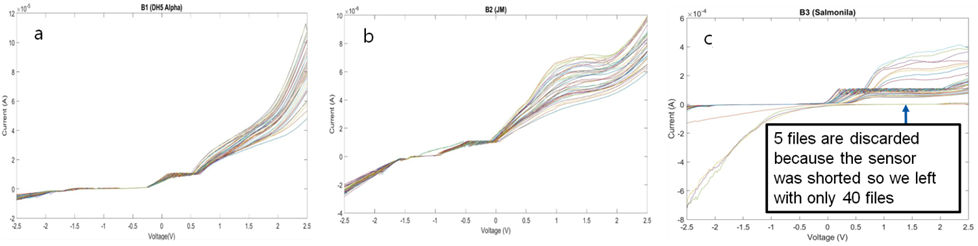


**Figure S8. The measured current against ±2.5 V for (a) *DH5-α*, (b) *JM109,* and (c) *Salmonella typhimurium*.**

- 1. **Data set and feature representation**


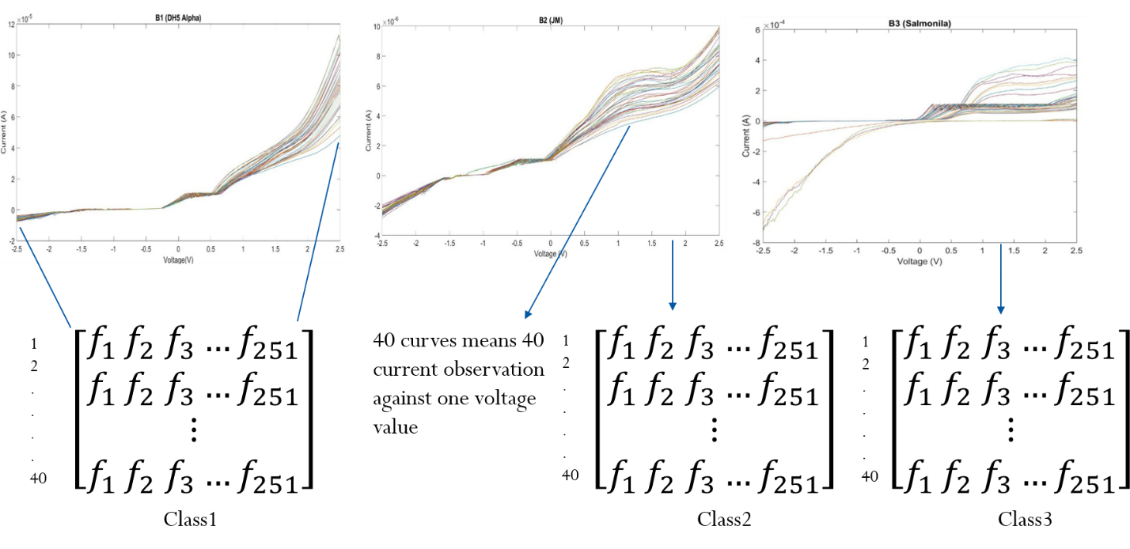


**Figure S9. Data extraction and representation.**

There are 45 I-V measurements curves (45 observation for each current point in the graph) for each bacteria as shown in Figure S9. There are 251current values or points against voltage sweep of ±2.5 V, which represented the characteristics of each bacteria. First, we have arranged the measured data into matrix form as shown in Figure S9. Here,$f_{i}$ represents the$i^{th}$ feature, which is $i^{th}$ measured current point at $i^{th}$ applied voltage. To extract the features, we took first, second derivatives and power calculation using the measured current from the sensor as shown in Figures 10a and 10b.


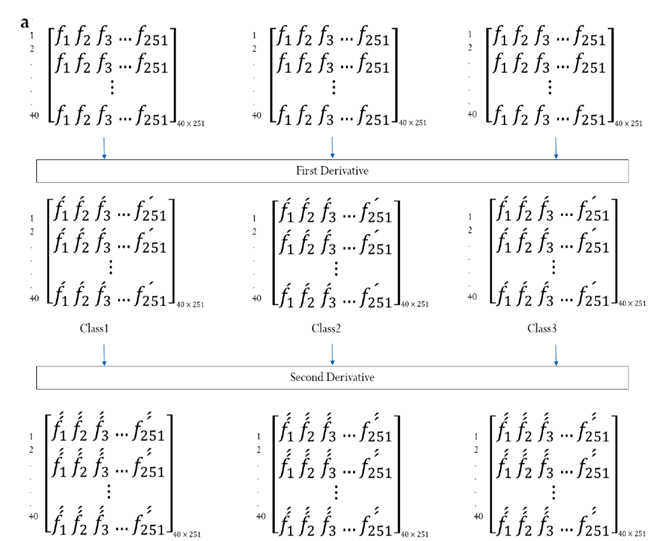


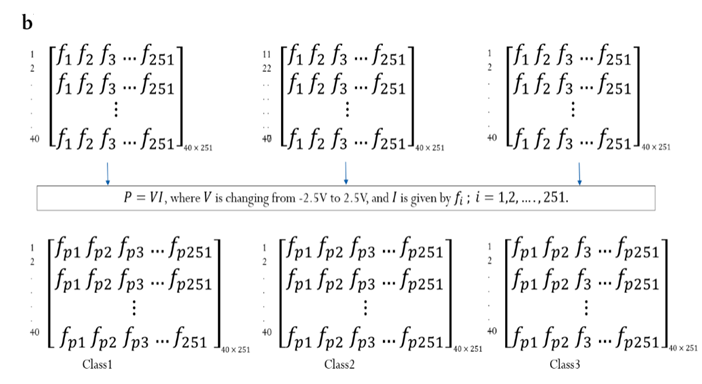


**Figure S10. Features extraction from measured data, (a) first and second derivative and (b) power.**

Data for the first and second derivative of current is graphically shown in Figure S11. In this figure, three classes C_1_, C_2_, and C_3_ represent *E. coli strains* DH5-α, JM109, and *Salmonella*, respectively. The extracted features from the measured data are used in machine algorithms to classify the bacteria type.


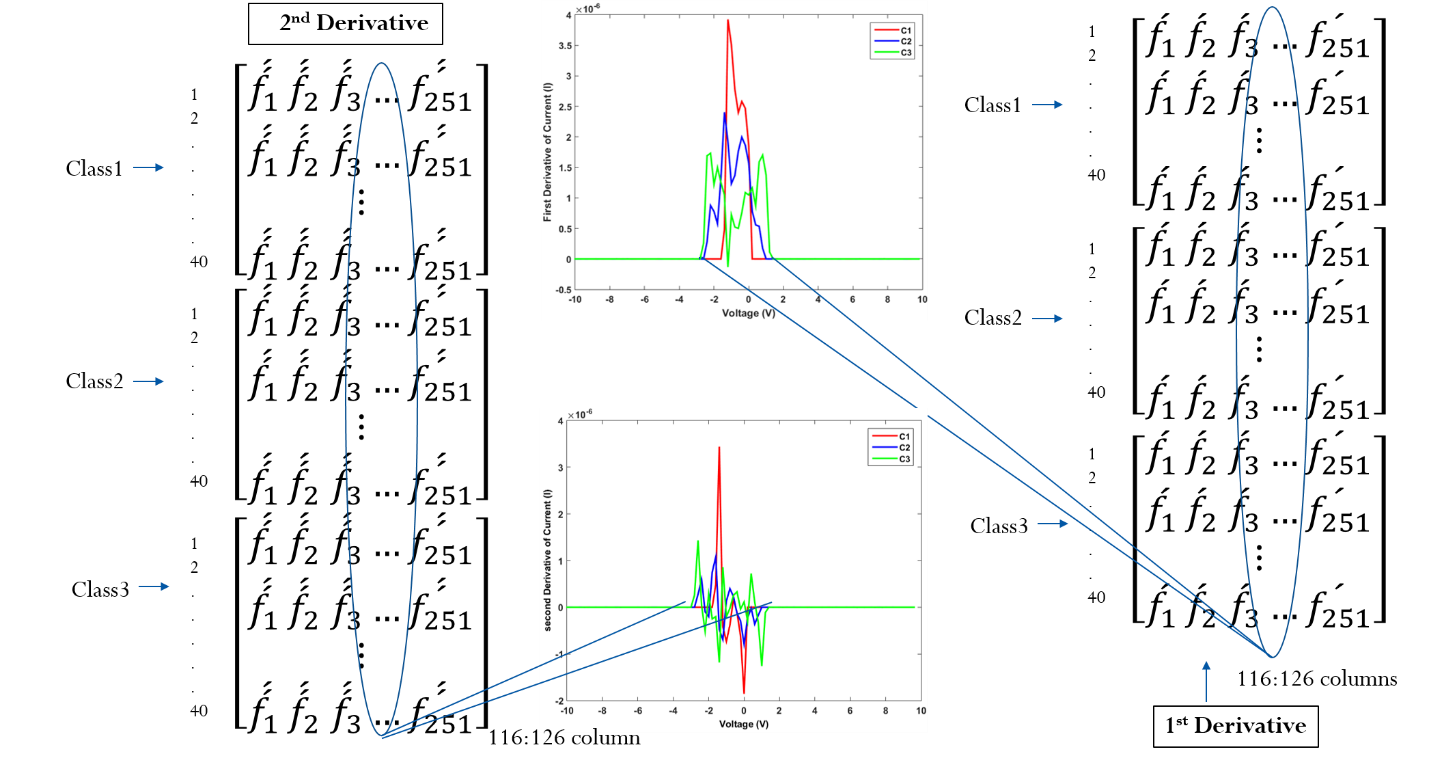


**Figure S11. Graphically representation of features; first and second derivate of measured current for three classes of bacteria *E. coli* strains DH5-α, JM109, and *Salmonella*.**

- 1. **Classification using algorithms**

To evaluate the classification accuracy, several linear and nonlinear data analysis techniques were selected in the paper. The linear and nonlinear classifiers can be used depending upon the requirements; if detection accuracy is of vital importance than training time of classifier, then nonlinear classifiers are usually applied. On the contrary, the linear classifier are faster than nonlinear due to the linear classifiers with linear kernels faster and simpler than nonlinear kernels in the non-linear classifiers. Therefore, if accuracy is not concerned parameter and fast training is more important, the linear classifier are preferred. Moreover, the linear classification methods can only solve classification problems that are linearly separable (usually via a hyper plane). The linear methods are preferred because these involve only linear combinations of data that leads to easier implementation of an algorithm. The nonlinear methods use nonlinear functions of data (the activation function in NN) and involve transformation of input dataset to a more stable space. Most of the time, this transformation describes the data features in more clear structure in comparison with the original space. For this reason, the classification algorithms can create more accurate prediction in a new stable space. After this transformation, the nonlinear methods were used linear method for separation. If the problem was extended to more than 3 classes, we have to consider whether the accuracy is important or the time consumption. To verify the proposed biosensor, the classifiers of both methods are applied as following subsections.

- - 1. **Maximum Likelihood Estimation (MLE)**

First, linear classification algorithm MLE has been applied to the measured data set. The following procedure has been adopted to classify the bacteria types:

- Calculate mean and covariance for each class
- Create ML model for each class
- Pass that ML model and mean of each class to Bayesian classifier
- Plot the data using scatter command
- Plot the line based on mean and covariance of each class data
- The Bayesian classifier returns the posterior on which class labels are decided

From features, we took two features of each bacteria class for ML classification as shown in Figure S12. The ML algorithm achieved 100% classification accuracy with two features as shown in Figure S12. As we can obtained two features are valuable, therefore, the other corresponding features (1^st^ derivative, 2^nd^ derivative, and power etc.) are also better. The accuracy of the classifier is very high because of good features. By using other features, the accuracy degrades. The mean of class1 (C_1_), class2 (C_2_), and class 3 (C_3_) are given as:

| Class Name | Mean (µ) |
| --- | --- |
| C1 (Red) | $\left( -3.6854\times{10}^{-6}, -1.4572\times{10}^{-6} \right)$ |
| C2 (Blue) | $\left( -1.4052\times{10}^{-6}, -0.5001\times{10}^{-6} \right)$ |
| C3 (Green) | $\left( -6.3884\times{10}^{-6}, -2.4175\times{10}^{-6} \right)$ |

By using mean and covariance the decision, we have computed discriminant functions (based on likelihood) of each class. To find the decision boundary between the classes, the discriminant functions are compared against each other. The decision boundary between class1 and class 2 is given by define by$g_{1,2}\left( x \right)$. The decision boundaries between class 1 and class 3, and class 2 and class 3 are given as $g_{1,3}\left( x \right)$ and $g_{2,3}\left( x \right)$, respectively.

$$g_{1,2}\left( x \right)=2.19\times{10}^{12}{x_{1}}^{2}+5.4\times{10}^{12}x_{1}x_{2}-1.25\times{10}^{7}x_{1}+4.2 \times{10}^{12}{x_{2}}^{2}+7.03\times{10}^{6}x_{2}-51.29$$

$$g_{1,3}\left( x \right)=3.34\times{10}^{13}{x_{1}}^{2}+1.05\times{10}^{14}x_{1}x_{2}-7.09\times{10}^{8}x_{1}+5.39 \times{10}^{14}{x_{2}}^{2}+3.29\times{10}^{9}x_{2}-6289.15$$

$$g_{2,3}\left( x \right)=3.12\times{10}^{13}{x_{1}}^{2}+1.004\times{10}^{14}x_{1}x_{2}-7.28\times{10}^{8}x_{1}+5.35 \times{10}^{14}{x_{2}}^{2}+3.28\times{10}^{9}x_{2}-6340.447$$

In the above equations, $x_{1}$ and $x_{2}$ are features of classes.

**
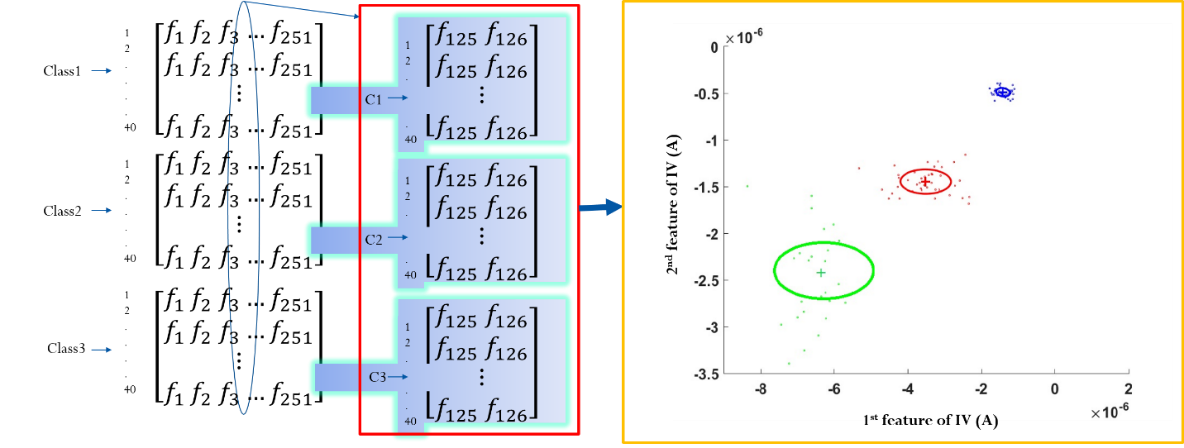
**

**Figure S12.The MLE algorithm application on the features of three classes of bacteria *E. coli* strains DH5-α (red), JM109 (blue), and *Salmonella (green)*. The bacteria classes are clearly separable on the basis on their features.**

- - 1. **Neural Network (NN)**

The feedback neural network is a nonlinear classifier and its learning process depends on adjusting the weights by using three types of samples: training, validation and testing. By using the error (which resulted after an iteration), the training samples support the neural network in its learning process of adjusting the weights. Neural networks can approximate any continuous function. Hence, they can provide nonlinear models allowing efficient predictions.

A neural network has three layers: input layer, hidden layer, and output layer. Each layer contains a number of neurons, which is interconnected with each other. The neuron activity implies a function that is named as an activation function. The activation function is applied to a linear combination of input values to which a constant value called bias or threshold, is added. In general, the activation functions are the sigmoid function and step function. An important step of a neural network algorithm is the learning process, which is to adjust the weights of the network. One of these methods is based on gradient method that estimates the optimal size of weights and ensures the local minimum of a function. These weights help the neural network to accelerate the learning process because in point of fact, their information are in the weights, not in the neurons.

Non-linear back propagation Neural Network (BPNN) has been applied by considering all features of bacteria types as shown in Figure S13. The input is consisted of 251 features and, 10 nodes are selected in hidden layer while 3 nodes in output (as there are three classes of bacteria). The data is divided randomly among training (70%), validation (15%) and testing (15%) data sets which are 84, 18 and 18 samples respectively. The back propagation training algorithm is scaled conjugate gradient and the performance has been evaluated by cost function of cross-Entropy as this is multiclass classification problem. The cross-entropy function attains its convergence threshold in 131 epochs and all samples (training, validation and testing) were identified with 0% error, as shown in Figure S14.

**
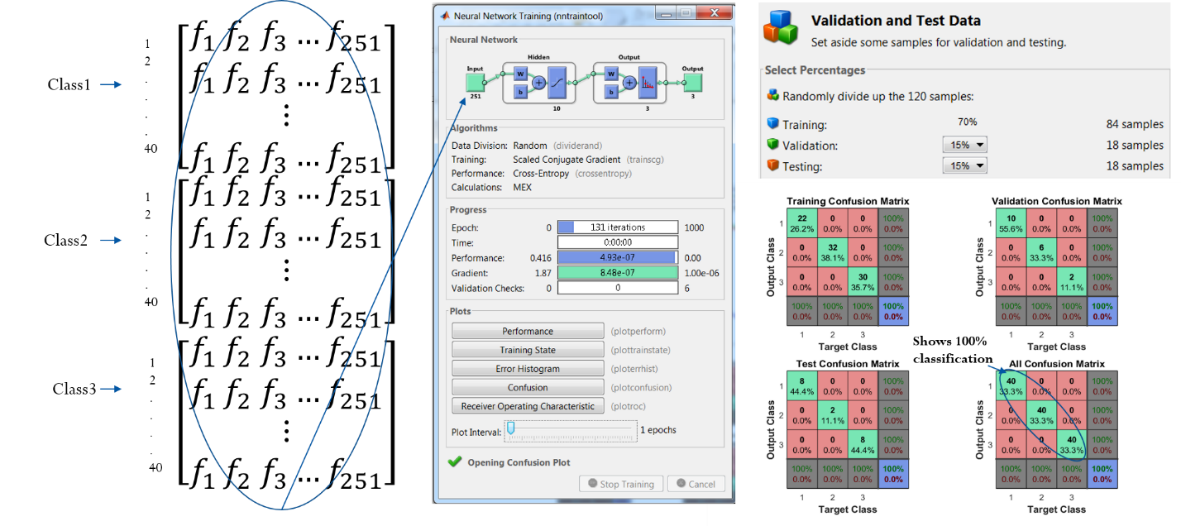
**

**Figure S13. The non-linear back propagation Neural Network algorithm application on all the features of three classes of bacteria *E. coli* strains DH5-α, JM109, and *Salmonella*. All 40 samples of each class correctly classified.**

The learning process for a neural network means adjusting the weights by using three types of samples: training, validation, and testing. A special principle is that the training samples are used to support neural network in its process of adjusting weights by using the error resulted after an iteration has been made. Neural networks are able to approximate any continuous function and that is why they can provide nonlinear models for time series allowing some efficient predictions.

**
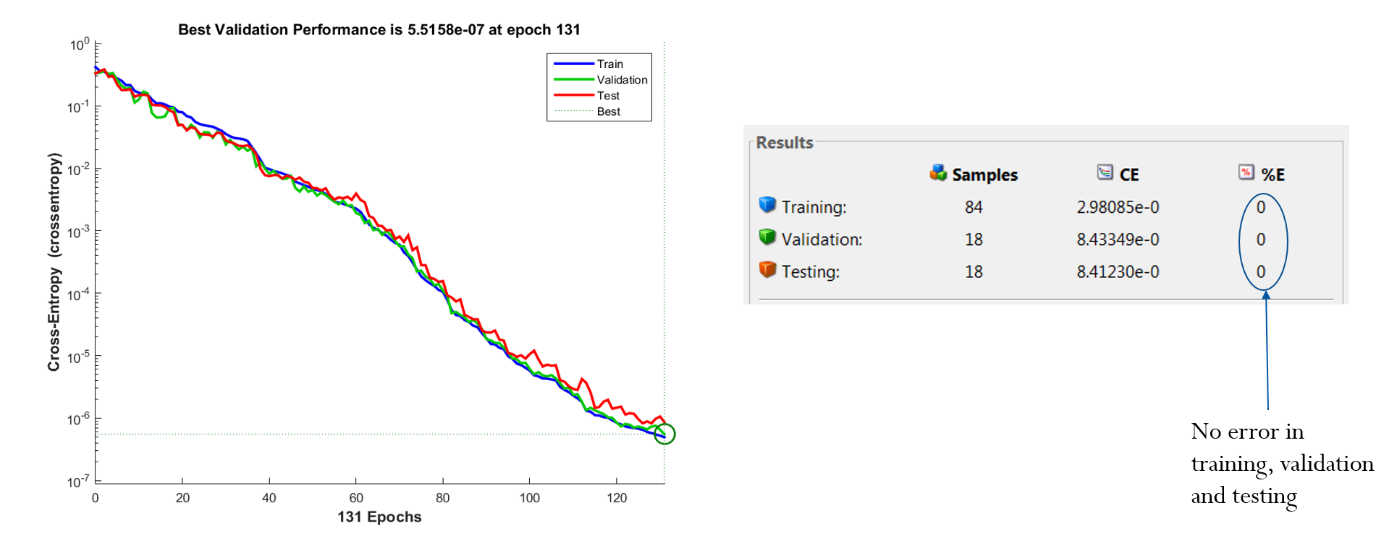
**

**Figure S14. The convergence of Neural Network algorithm in training, validation and testing. The training, validation and testing data samples are classified with 0% error.**

- - 1. **Linear Discriminant Analysis (LDA)**

The LDA algorithm has been applied to the features of all three classes of bacteria *E. coli* strains DH5-α, JM109, and *Salmonella* as shown in Figure S15. For LDA, we have assumed the data to be normally distributed as the distribution of values generally cluster around an average. Here, only one feature set current (I) has statistically independent and the other features, power, first and second derivatives have dependent behavior. Our all-data sets do not fit to the ideal conditions of LDA. However, the current feature set fulfills the LDA conditions due to it is statistically independent for each class. We assume covariance matrices are identical for classes because current features have identically shaped clusters about their mean vectors. First, we used approach; "one against the rest" in which the points from class 1 class are put in one group and consider, everything else in the other group, and then applied the LDA applied. We can see that in first step, the class 1 (JM109) and class 2 (DH5-α) are separated and then in next step, class 1 and class 3 (*Salmonella*) are separated by a separation line. While in the third step, boundary decision is made between class 2 and class 3. We can see that LDA classified the problem 100% correctly. The decision boundary equation between class 1 and 2 is given by:$-25.9838 + 6.23727\times{10}^{6}x+ 5.29451\times{10}^{6}y=0$. The decision boundary equation between class 1 and 3 is $86.515 -9.48098\times{10}^{6}x+ -7.17934e\times{10}^{6}y =0$ and the decision boundary equation between class 2 and 3 is represented by following polynomial; $112.499-1.57183\times{10}^{7}x-1.24739\times{10}^{7}y =0$.Where $x$ and $y$ are the features.

**
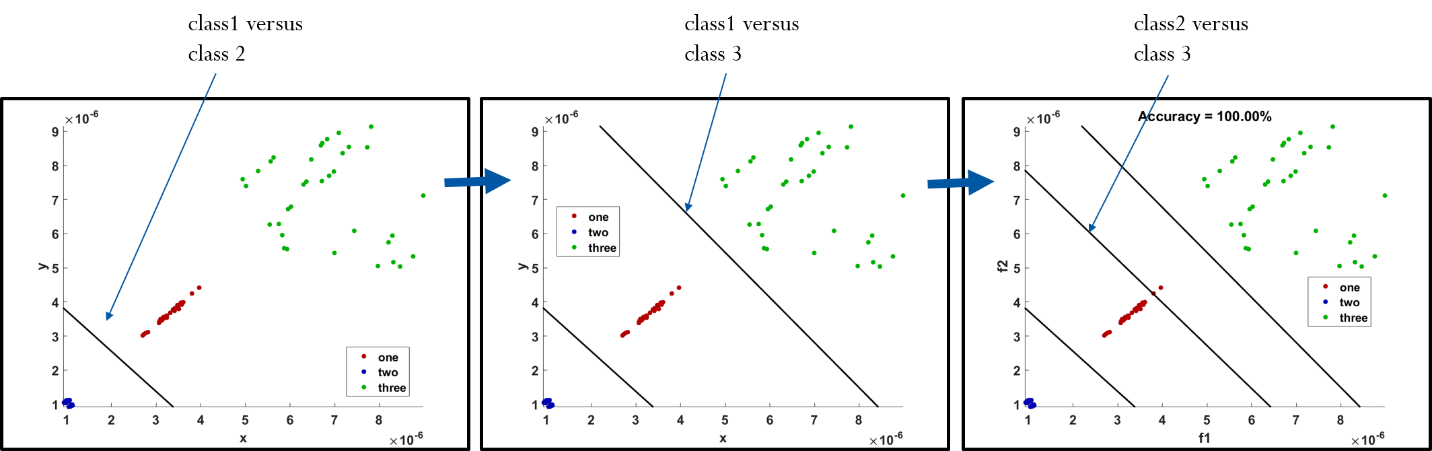
**

**Figure S15. Step by step application of LDA on the features of three classes of bacteria; *E. coli* strains DH5-α, JM109, and *Salmonella*.**

- - 1. **Conclusion of classification**

To evaluate the performance of multi-class classification problem, linear and non-linear algorithms have been applied by using the measurement data obtained from the proposed biosensor. Due to we can got the good features from the proposed biosensor, the above results indicates that 100% efficiency can be achieved using the various ML, Neutral Network (BPNN), and LDA. Table S1shows the applied algorithms with the classification accuracy.

Table S1. Performance of Machine Algorithms.


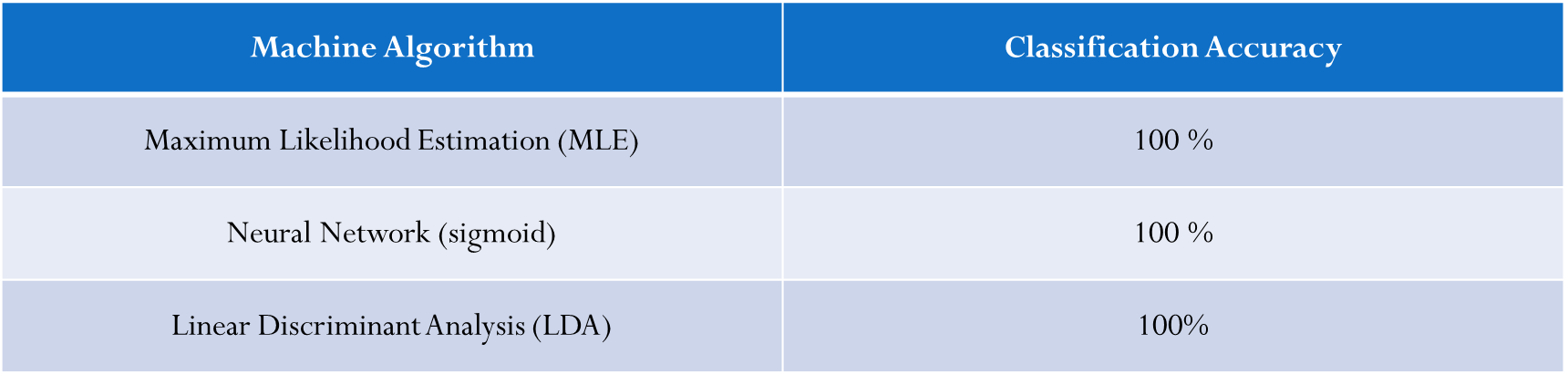

Supplement: Supplementary file 1 — Supplementary information [file 41598_2018_24208_MOESM1_ESM.docx]
